# SUMOylation of IGF2BP2 promotes vasculogenic mimicry of glioma via regulating OIP5-AS1/miR-495-3p axis

**DOI:** 10.7150/ijbs.58035

**Published:** 2021-07-13

**Authors:** Hao Li, Di Wang, Bolong Yi, Heng Cai, Yipeng Wang, Xin Lou, Zhuo Xi, Zhen Li

**Affiliations:** 1 Department of Neurosurgery, Shengjing Hospital of China Medical University, Shenyang, China; 2 Liaoning Clinical Medical Research Center in Nervous System Disease, Shenyang, China; 3 Key Laboratory of Neuro-oncology in Liaoning Province, Shenyang, China

**Keywords:** SUMOylation, IGF2BP2, OIP5-AS1, miR-495-3p, vasculogenic mimicry

## Abstract

**Rationale:** Glioma is the most common primary malignant tumor of human central nervous system, and its rich vascular characteristics make anti-angiogenic therapy become a therapeutic hotspot. However, the existence of glioma VM makes the anti-angiogenic therapy ineffective. SUMOylation is a post-translational modification that affects cell tumorigenicity by regulating the expression and activity of substrate proteins.

**Methods:** The binding and modification of IGF2BP2 and SUMO1 were identified using Ni^2+^-NTA agarose bead pull-down assays, CO-IP and western blot; and in vitro SUMOylation assays combined with immunoprecipitation and immunofluorescence staining were performed to explore the detail affects and regulations of the SUMOylation on IGF2BP2. RT-PCR and western blot were used to detect the expression levels of IGF2BP2, OIP5-AS1, and miR-495-3p in glioma tissues and cell lines. CCK-8 assays, cell transwell assays, and three-dimensional cell culture methods were used for evaluating the function of IGF2BP2, OIP5-AS1, miR-495-3p, HIF1A and MMP14 in biological behaviors of glioma cells. Meantime, RIP and luciferase reporter assays were used for inquiring into the interactions among IGF2BP2, OIP5-AS1, miR-495-3p, HIF1A and MMP14. Eventually, the tumor xenografts in nude mice further as certained the effects of IGF2BP2 SUMOylation on glioma cells.

**Results:** This study proved that IGF2BP2 mainly binds to SUMO1 and was SUMOylated at the lysine residues K497, K505 and K509 sites, which can be reduced by SENP1. SUMOylation increased IGF2BP2 protein expression and blocked its degradation through ubiquitin-proteasome pathway, thereby increasing its stability. The expressions of IGF2BP2 and OIP5-AS1 were up-regulated and the expression of miR-495-3p was down-regulated in both glioma tissues and cells. IGF2BP2 enhances the stability of OIP5-AS1, thereby increasing the binding of OIP5-AS1 to miR-495-3p, weakening the binding of miR-495-3p to the 3'UTR of HIF1A and MMP14 mRNA, and ultimately promoting the formation of VM in glioma.

**Conclusions:** This study first revealed that SUMOylation of IGF2BP2 regulated OIP5-AS1/miR-495-3p axis to promote VM formation in glioma cells and xenografts growth in nude mice, providing a new idea for molecular targeted therapy of glioma.

## Introduction

Glioma accounts for about 80% of human primary brain tumors, which is the most common central nervous system tumor 1. The characteristics of high recurrence rate make the average median survival period of glioma patients only about 12-15 months 2. Ascribable to the rich vascularity of glioma, anti-angiogenic therapy has become a new treatment scheme which has attracted much attentions. However, this treatment scheme does not significantly improve the survival rate of patients 3. The reason may be the vasculogenic mimicry (VM) in glioma, which is a tubular structure formatted by highly plastic multipotent malignant tumor cells. This special microcirculation system provides blood supply for tumors and involved in the migration and invasion of tumor cells 4, 5. Many studies have demonstrated that VM is widely existed in various types of malignant tumors such as human gastric cancer, non-small cell lung cancer, melanoma, liver cancer, and glioma 6-10. In glioma, VM has closely correlation with the pathological grade, proliferation, migration and invasion of tumor 11, 12.

SUMOylation is one of the post-translational modifications (PTMs) of proteins, which is participated in the regulation of multiple pivotal biological functions and has closely correlation with the tumorigenesis and development of the tumors 13-16. The essence of SUMOylation is a biological modification process of small molecule ubiquitin-like modifier (SUMO) attached to the protein substrate. The main form of SUMOylation is the binding of SUMO molecules to the receptor lysine residue of the target protein, and the substrate modification is gradually carried out through three enzymatic cascade steps [including the activation of heterodimer E1 enzyme (SAE1 and SAE2/UBA2) involved, the conjugation of E2 enzyme Ubc9mediated and substrate modification through the co-operation of the E2, E3 protein ligases], thereby regulating the structure and function of the substrate protein. In addition, SUMOylation is a dynamic and reversible process. SUMO-specific proteases (SENPs) can specifically de-SUMOylated the substrate protein, and cooperate with SUMO molecules to regulate the SUMOylation state of the substrate protein, thereby regulating cell function 14, 17. Many studies have shown that SUMOylation may affect the expression, stability and subcellular localization of substrate proteins 18, 19, but its regulatory mechanism in tumorigenesis is still unclear.

Insulin-like growth factor 2 mRNA-binding protein 2 (IGF2BP2), one member of the IGF2 mRNA-binding protein family, is encoded by the IGF2BP2 gene which located on chromosome 3q27.2 20. It plays an essential role in regulating subcellular mRNA localization, stability and translation 21, 22. Extensive studies have shown that IGF2BP2 is participated in regulating tumorigenesis. For example, in pancreatic cancer, up-regulation of IGF2BP2 promotes tumor cell proliferation by involved the PI3K/Akt signaling pathway activation 23. Overexpression of IGF2BP2 promotes the progression of pancreatic cancer cells, which can be treated as a marker for the diagnosis and prognosis of pancreatic cancer 24. In the nervous system, knockdown of IGF2BP2 can significantly reduce FBXL19-AS1 and tight junction related proteins expression, and then promote the permeability of blood-tumour barrier via STAU1-mediated mRNA decay negatively regulating ZNF765 expression 25. At present, the study of IGF2BP2 on VM of glioma has not been reported.

LncRNAs are a class of non-coding RNA with limited coding protein capability which length are more than 200 nucleotides. It plays an essential role in regulating tumorigenesis and development of the tumors 26. Current study has shown that OIP5-AS1 was up-regulated in breast cancer which regulates the expression of ZEB2 by acting as competitive endogenous RNA (ceRNA) for miR-340-5p, and then promotes metastasis of breast cancer 27. Similarly, OIP5-AS1 regulated ovarian cancer progression via miR-137/ZNF217 axis 28. Knockdown of OIP5-AS1 up-regulates miR-410 to specifically block Wnt-7b/β-catenin pathway, thereby inhibiting the growth, invasion and migration of glioma cells, and promoting their apoptosis 29. However, the effects of OIP5-AS1 on the VM formation ability of glioma cells, remain unclear.

MiRNAs, a class of endogenous small non-coding RNAs with a length of about 20-22 bps, which are mainly participated in the post-transcriptional gene expression regulation by binding to the 3'UTR of the target gene, causing the degradation or inhibition of mRNAs, and thus playing an important role in the regulation of cell development, proliferation, apoptosis and differentiation 30, 31. MiR-495-3p is a key regulator of tumorigenesis and malignant progression which expression is dysregulated in varieties of tumors. MiR-495-3p expression in prostate cancer is down-regulated, and the expression of TRIP13 is regulated by competitive combination with NORAD, which affects the proliferation, migration, invasion and apoptosis of tumor 32. MiR-495-3p inhibits tumor cell proliferation, migration and invasion by targeting CTRP3 in osteosarcoma 33. In glioma, LGMN acts as a sponge of miR-495-3p through ceRNA mechanism to promote tumor progression 34. Nevertheless, the regulation mechanism of miR-495-3p in VM capacity of glioma still unclear.

HIF1A is one of the HIF complexes, which plays a crucial role in regulating oxygen homeostasis in physiological and pathological environment. HIF1A has been confirmed to be involved in the formation of VM in colorectal cancer and salivary adenoid cystic carcinoma, and has become a marker protein of VM and indicated poor prognosis of tumor 35, 36.

Matrix metalloproteinase 14 (MMP14) belongs to the family of metalloproteinases that degrade extracellular matrix. Recent studies have shown that MMP14 is one of VM marker proteins which positively correlated with VM formation in lung cancer and glioma 11, 37. In glioma, whether miR-495-3p regulates the expression of HIF1A and MMP14 or the formation of VM has not been reported.

In this study, we found that K497/505/509R was the main SUMOylation site of IGF2BP2, which could be SUMOylated mainly combined with SUMO1 and be de-SUMOylated by SENP1. SUMOylation can increase the expression of IGF2BP2 protein, block its degradation and then increase its stability, but has no effect on subcellular localization of IGF2BP2. The mutation of SUMOylation site significantly inhibited the SUMOylation of IGF2BP2, thereby reducing the VM capacity of glioma cells. We further detected the endogenous expression levels of IGF2BP2, OIP5-AS1, and miR-495-3p in both glioma tissues and cells, and further explored the mutual regulatory mechanism between these molecules and their effects on VM capacity of glioma cells. SUMOylation increased the expression and stability of IGF2BP2, and further promoted VM capacity of glioma cells via OIP5-AS1/miR-495-3p axis. In vivo experiments also confirmed that SUMOylation promoted the growth of xenograft tumors in nude mice. This study intends to provide new evidence for revealing the mechanism of glioma pathogenesis and development, and to find new targets for glioma molecular therapy.

## Results

### IGF2BP2 mainly combined with SUMO1 to form SUMO modification

**Figure 1:**
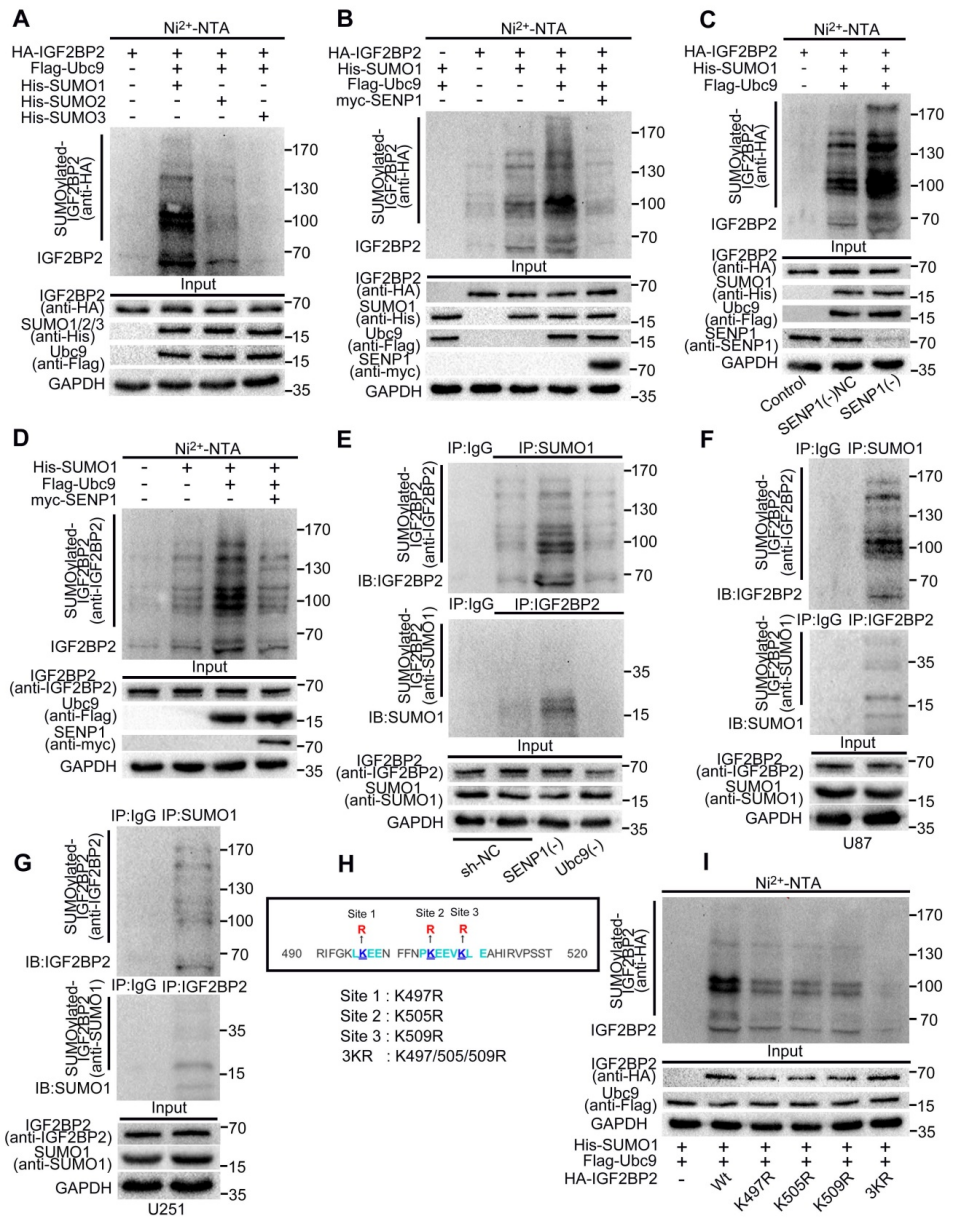
** IGF2BP2 is mainly modified by SUMO1, and K497R, K505R and K509R are the major SUMOylation sites in IGF2BP2. (A)** IGF2BP2 is mainly modified by SUMO1 in HEK293T cells. HEK293T cells were transfected with HA-IGF2BP2, Flag-Ubc9 and His-SUMO1, His-SUMO2 or His-SUMO3, then the lysates were subjected to purification and precipitation with Ni^2+^-NTA agarose bead pull-down assay, and followed by western blot with indicated antibodies. **(B)** The SUMOylation of IGF2BP2 can be enhanced by Ubc9 and removed by SENP1. HEK293T cells were transfected with indicated plasmids (HA- IGF2BP2, with or without His-SUMO1, Flag-Ubc9 and myc-SENP1) and the SUMOylation were detected by Ni^2+^-NTA agarose bead pull-down assay. **(C)** The SUMOylation of IGF2BP2 can be enhanced by the knockdown of SENP1. SENP1 was knocked-down in HEK293T cells, while HA-IGF2BP2, Flag-Ubc9 and His-SUMO1 were co-transfected into HEK293T above. The lysates were subjected to precipitation with Ni^2+^-NTA agarose bead pull-down assay, and followed by western blotting with indicated antibodies. **(D)** Endogenous IGF2BP2 is modified by SUMO1. HEK293T cells were transfected with indicated plasmids (His-SUMO1, with or without Flag-Ubc9 and myc-SENP1), anti-IGF2BP2 antibody was used to detected the SUMOylated of IGF2BP2 bands by following the Ni^2+^-NTA agarose bead pull-down assay. **(E)** Endogenous IGF2BP2 can be SUMOylated by endogenous SUMO1 in HEK293T cells, confirmed by CO-IP method. HEK293T cells were transfected with indicated plasmids [sh-NC, SENP1(-) and Ubc9(-)], then lysed for CO-IP method with anti-SUMO1 antibody or anti-IGF2BP2 antibody or normal IgG, followed by western blot assay with anti-IGF2BP2 and anti-SUMO1 antibodies. **(F-G)** SUMOylation of endogenous IGF2BP2 naturally occurs in giloma cells (U87 and U251). Cell Lysates from U87 and U251 were used for CO-IP method with anti-SUMO1 antibody or anti-IGF2BP2 antibody or normal IgG, followed by western blot assay with anti-IGF2BP2 and anti-SUMO1 antibodies. **(H-I)** K497R, K505R and K509R are the major SUMOylation sites in IGF2BP2. (H) The main SUMO-sites (K497R, K505R and K509R) for SUMOylation predicted by bioinformatics software. (I) The mutant 3KR (K497/505/509R) reduces the SUMOylation of IGF2BP2 most. HEK293T cells were transfected with indicated plasmids (HA-IGF2BP2-Wt or different IGF2BP2 mutants (K497R, K505R, K509R and 3KR) and His-SUMO1/Flag-Ubc9), Lysates were purified by Ni^2+^-NTA agarose bead pull-down assay, and followed by western blotting with indicated antibodies.

In order to determine whether IGF2BP2 can be SUMOylated in cells, HA-IGF2BP2, His-SUMO1, His-SUMO2, His-SUMO3 and Flag-Ubc9 were respectively transfected into HEK293T cells. Ni^2+^-NTA agarose bead pull-down assays were used to pull down the His-SUMO conjugates for Western blotting. Our results showed that IGF2BP2 could be modified by SUMO1, SUMO2 and SUMO3, and the modification by SUMO1 was the strongest (Fig. 1A). Therefore, the follow-up studies focused on the modification effect between IGF2BP2 and SUMO1.

SENP1 is one of the SUMO specific proteases and participates in the process of SUMO modification. This study continues to verify the effect of SENP1 on SUMOylation of IGF2BP2. We transfected HA-IGF2BP2, His-SUMO1, Flag-Ubc9 and myc-SENP1 into HEK293T cells, respectively. Ni^2+^-NTA agarose bead pull-down assays were used to verify if SENP1 can remove the SUMO1 modification. We found that Ubc9 significantly increased the SUMO1 modification of IGF2BP2, while SENP1 significantly decreased the modification effect above, which played a role of de-SUMOylation (Fig. 1B).

In addition, in this study, plasmid of SENP1 knockdown was stably transferred to HEK293T cells, and the SUMO1 modification of IGF2BP2 was further verified by Ni^2+^-NTA agarose bead pull-down assays. Compared with NC group, the SUMOylation of IGF2BP2 in SENP1(-) group was significantly enhanced (Fig. 1C).

His-SUMO1, Flag-Ubc9 with or without SENP1 were co-transfected into HEK293T cells. Ni^2+^-NTA agarose bead pull-down assays were used to verify whether endogenous IGF2BP2 can combined with SUMO1 and be SUMOylated by SUMO1. The SUMOylation bands were detected by using anti-IGF2BP2 antibody, and SUMO1 modification was the best in the presence of Flag-Ubc9, but could almost be removed by SENP1 (Fig. 1D).

In this study, co-immunoprecipitation (CO-IP) was used to further verify the binding and modification of endogenous SUMO1 on endogenous IGF2BP2. First, HEK293T cells were stably transfected with knockdown SENP1, knockdown Ubc9 and sh-NC plasmid respectively, and then, the lysates were immunoprecipitated with SUMO1, IGF2BP2 or normal IgG antibodies. Finally, western blot was performed with anti-IGF2BP2 and anti-SUMO1 antibodies. The results showed that the endogenous SUMO1 could bind and modify the endogenous IGF2BP2 in HEK293T cells. The SUMOylation of IGF2BP2 was enhanced by SENP1 knockdown, while the SUMOylation of IGF2BP2 was significantly inhibited by Ubc9 knockdown. Compared with control group, input group confirmed that the knockdown of SENP1 and Ubc9 did not affect IGF2BP2 expression (Fig. 1E).

To further deepen understanding the mutual modification of endogenous IGF2BP2 and endogenous SUMO1 in cells. Lysates of U87 and U251 cells were used for IP with anti-SUMO1, anti-IGF2BP2 and anti-IgG antibodies, and then western blot was performed with anti-IGF2BP2 and anti-SUMO1 antibodies. The results indicated that endogenous IGF2BP2 could bind to endogenous SUMO1 in glioma cells (U87 and U251) and undergo SUMOylation (Fig. 1F-G).

### K497, k505 and k509 are the main SUMO-sites of IGF2BP2

In order to determine the main SUMO-sites of IGF2BP2 in human, we predicted that lysines (Ks) at K497, k505 and k509 could be mutated to arginine (R) according to the analysis results of bioinformatics software, and then as the main SUMO-sites for SUMOylation identification (Fig. 1H). we mutated the predicted lysins of IGF2BP2 protein, then co-transfected three single-lysin mutants and triple-lysin mutant with plasmids His-SUMO1 and Flag-Ubc9 into HEK293T cell for SUMO modification analysis by Ni^2+^-NTA agarose bead pull-down assays. The results indicated that compared with the wild-type (-Wt), the SUMO modification levels of IGF2BP2 were decreased in K497R, K505R, K509R and 3KR (K497/505/509R), and the SUMO modification level decreased most significantly in 3KR (K497/505/509R) (Fig. 1I).

### SUMOylation enhanced the stability of IGF2BP2 by repressing the ubiquitin-proteasome pathway, but did not affect its subcellular localization

**Figure 2:**
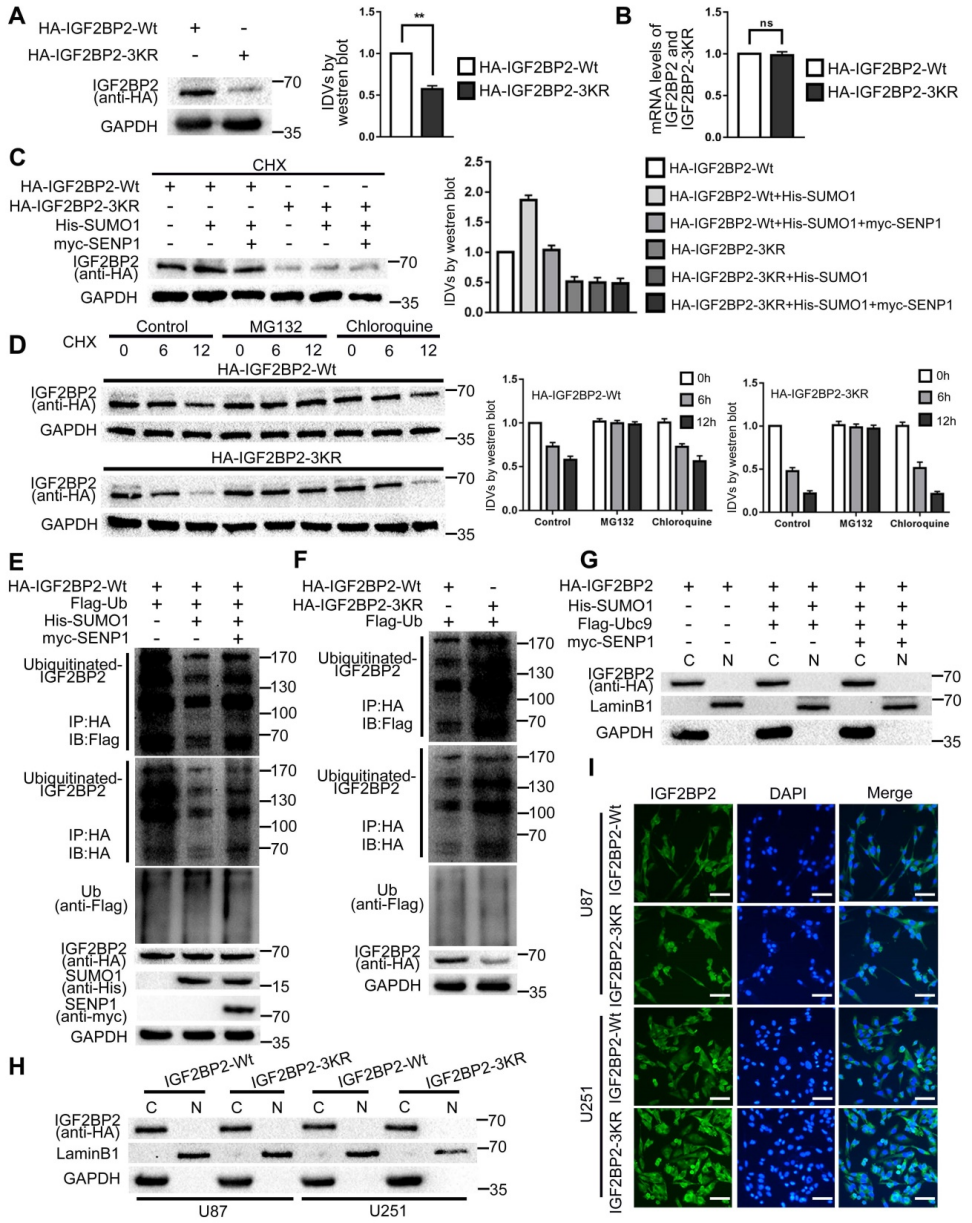
** SUMOylation enhanced IGF2BP2 expression and stability, but did not affect its subcellular localization. (A-B)** HA-IGF2BP2-3KR mutant reduce the protein expression of IGF2BP2. (A) HEK293T cells were transfected with HA-IGF2BP2-Wt or HA-IGF2BP2-3KR, Lysates were detected by western blotting with anti-HA antibody. Data are presented as the mean ± SD (n=3), **P < 0.01, vs. HA-IGF2BP2-Wt. (B) RT-PCR was used to measure the mRNA level of IGF2BP2. Data are expressed as the mean ± SD (n=3). **(C)** SUMOylation increases the stability of IGF2BP2 but not the IGF2BP2-3KR mutant. HEK293T cells were transiently transfected with indicated plasmids (HA-IGF2BP2-Wt or HA-IGF2BP2-3KR, His-SUMO1 and myc-SENP1), after 24h of transfection, CHX (100μg/ml) was added to culture. Lysates were detected by western blotting with corresponding antibodies. **(D)** IGF2BP2 was degraded via the proteasome pathway, IGF2BP2-3KR exhibits faster degradation than IGF2BP2-Wt. HEK293T cells were transiently transfected with HA-IGF2BP2-Wt or HA-IGF2BP2-3KR, after 24h after transfection, CHX (100μg/ml) was added for 0 to 12 hours. Dimethyl sulfoxide (control group), MG132 (proteasome inhibitor, 20μM) or chloroquine (lysosome inhibitor, 100μM) were added to culture 1 hour before CHX. Lysates were detected by western blotting with corresponding antibodies. **(E)** SUMOylation of IGF2BP2 represses its ubiquitination. Lysates from HEK293T cells transiently transfected with HA-IGF2BP2-Wt, Flag-ubiquitin (Flag-Ub), His-SUMO1, or myc-SENP1 at various combinations as indicated were subjected to IP with anti-HA antibody under denaturing conditions, which was followed by IB with anti-Flag and anti-HA antibodies. The original lysates were also analyzed by IB for inputs of Flag-Ub, HA-IGF2BP2, His-SUMO1 and myc-SENP1. **(F)** The SUMOylation-sites mutant IGF2BP2-3KR has enhanced ubiquitination which compared to IGF2BP2-Wt. Similar to (E) but the cells were transfected with HA-IGF2BP2-Wt, HA-IGF2BP2-3KR, or Flag-Ub at various combinations as indicated. Note the stronger Flag-Ub labeling samples transfected with HA-IGF2BP2-3KR than HA-IGF2BP2-Wt. **(G-I)** SUMOylation did not change the subcellular localization of IGF2BP2. (G) HEK293T cells were transiently transfected with indicated plasmids (HA-IGF2BP2, His-SUMO1, Flag-Ubc9 and myc-SENP1), after the extraction of cytoplasmic and nuclear proteins, Lysates were detected by western blotting with corresponding antibodies. (H) U87 and U251 cells were transiently transfected with HA-IGF2BP2-Wt and HA-IGF2BP2-3KR, after the extraction of cytoplasmic and nuclear proteins, Lysates were detected by western blotting with corresponding antibodies, and (I) immunofluorescence staining was used to observed the subcellular localization of IGF2BP2. Scale bars: 100μm.

This study confirmed that SUMO modification could enhance the stability of IGF2BP2 by blocking the degradation process of IGF2BP2 in cells. First, the study found that there was no significant difference in the mRNA expression levels of HA-IGF2BP2-Wt and HA-IGF2BP2-3KR in HEK293T cells, but the protein expression level of HA-IGF2BP2-3KR was significantly lower than that of HA-IGF2BP2-Wt (Fig. 2A-B). Second, we co-transfected HA-IGF2BP2-Wt, HA-IGF2BP2-3KR, His-SUMO1 and myc-SENP1 into HEK293T cells, and used cycloheximide (CHX) to inhibit the synthesis of de novo proteins for 12 hours. We found that compared with the HA-IGF2BP2-Wt group alone, the expression level of IGF2BP2 protein in HA-IGF2BP2-Wt and His-SUMO1 co-transfected cells was significantly increased, but the protein expression enhancement effect was abolished when co-transfected with myc-SENP1 or HA-IGF2BP2-3KR (Fig. 2C). It is indicated that the degradation of IGF2BP2 was slowed down when conjugated with SUMO1. Third, the degradation of IGF2BP2 mainly occurs through ubiquitin-proteasome pathway. Compared with chloroquine (lysosomal inhibitor), MG132 (proteasome inhibitor) significantly blocked IGF2BP2 degradation in the presence of CHX (Fig. 2D). Moreover, the degradation time of HA-IGF2BP2-3KR group was shorter than that of HA-IGF2BP2-Wt group, and chloroquine did not block the degradation, while MG132 significantly blocked the degradation (Fig. 2D). To test how SUMOylation affects IGF2BP2 ubiquitination, we transfected HEK293T cells with plasmids for HA-IGF2BP2-Wt and Flag-ubiquitin (Flag-Ub). By using the immunoblotting assay with either anti-Flag or anti-HA antibody in immunoprecipitants pulled down by the anti-HA antibody, polyubiquitinated IGF2BP2 was detected (Fig. 2E). The co-expression of His-SUMO1 largely abolished IGF2BP2 ubiquitination under these conditions, and the effect of His-SUMO1 was reversed by co-expression of myc-SENP1 (Fig. 2E). Furthermore, when co-transfected with Flag-Ub in HEK293T cells, the SUMOylation-sites mutant HA-IGF2BP2-3KR shown more polyubiquitination than HA-IGF2BP2-Wt (Fig. 2F). These results indicate that SUMOylation represses IGF2BP2 ubiquitination, protecting IGF2BP2 from degradation by proteasomes. Based on the above results, SUMOylation can effectively protect the substrate protein IGF2BP2 from degradation by ubiquitin-proteasome pathway, thereby enhancing its stability.

In order to estimate if SUMOylation affects the subcellular localization of IGF2BP2, we co-transfected HA-IGF2BP2, His-SUMO1, Flag-Ubc9 and myc-SENP1 into HEK293T cells, and then the cytoplasmic and nuclear proteins were separated and extracted. Finally, the expression of IGF2BP2 protein was detected by western blot. The results showed that IGF2BP2 was mainly located in the cytoplasm, and SUMOylation did not affect the subcellular localization of IGF2BP2 (Fig. 2G). In this study, HA-IGF2BP2-Wt and HA-IGF2BP2-3KR were transfected into U87 and U251 cells, and the effect of SUMO-sites mutation on the subcellular localization of IGF2BP2 was detected by cytoplasmic and nuclear fractionation and Immunofluorescence staining. The results showed that after transfection of HA-IGF2BP2-Wt and HA-IGF2BP2-3KR, IGF2BP2 was still mainly located in the cytoplasm (Fig. 2H-I).

### IGF2BP2 and OIP5-AS1 were highly expressed in both glioma tissues and cells. The SUMO-site mutates (IGF2BP2-3KR), knockdown of IGF2BP2 and OIP5-AS1 inhibited the VM of glioma cells

**Figure 3:**
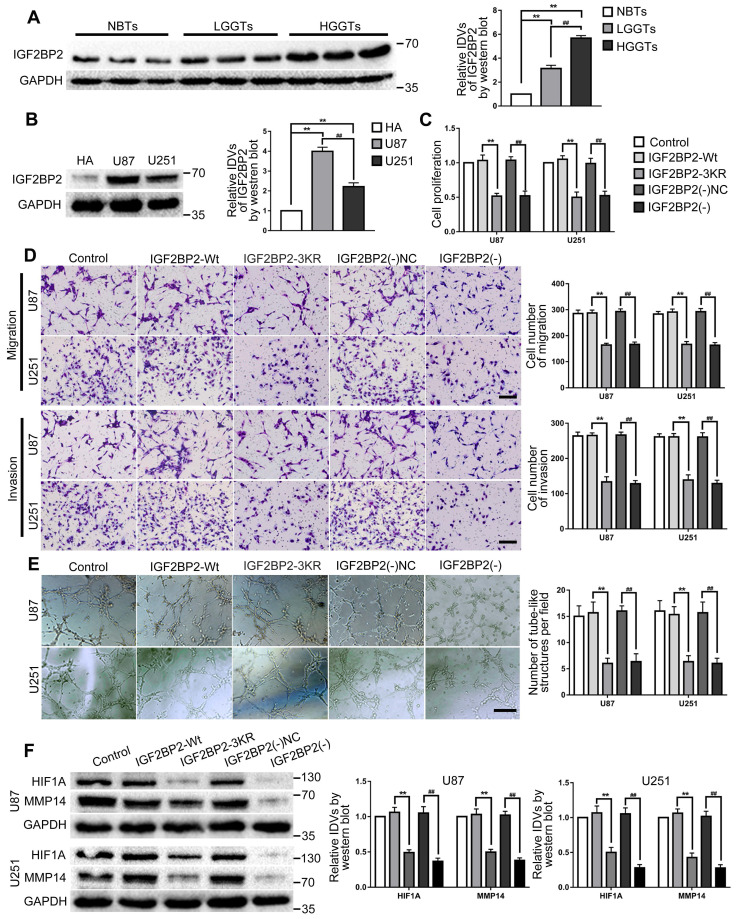
** IGF2BP2 and IGF2BP2 SUMOylation exerted tumor-promoting functions in glioma cells. (A)** Western blot was used to detected the protein expression levels of IGF2BP2 in NBTs, LGGTs and HGGTs. Data are presented as the mean ± SD (n=3), **P<0.01, vs. NBTs, ##P<0.01, vs. LGGTs. **(B)** Western blot was used to detected the protein expression levels of IGF2BP2 in HA cells, U87 cells and U251 cells. Data are presented as the mean ± SD (n=3), **P<0.01, vs. HA cells, ##P<0.01, vs. U87 cells. **(C-E)** CCK-8 assay, cell transwell assay, and three-dimensional cell culture method were used to detect the effects of IGF2BP2 knockdown and IGF2BP2-3KR on proliferation, migration, invasion and VM formation. Data are presented as the mean ± SD (n=3), **P<0.01, vs. IGF2BP2-Wt, ##P<0.01, vs. IGF2BP2(-)NC. Scale bar: 50μm. **(F)** Western blot was used to detected the protein expression levels of HIF1A and MMP14 in glioma cells (U87 and U251) after knockdown IGF2BP2 and IGF2BP2-3KR mutant. Data are presented as the mean ± SD (n=3), **P<0.01, vs. IGF2BP2-Wt, ##P<0.01, vs. IGF2BP2(-)NC.

Western blot was used to detect the expression of IGF2BP2 in glioma tissues and cells. The results showed that in comparison to the NBTs, IGF2BP2 expression in LGGTs and HGGTs were significantly increased, and the expression increased as the pathological grade level increased; while IGF2BP2 expression in U87 and U251 cells were significantly higher than that in HA cells (Fig. 3A-B). To further explore the potential mechanisms of IGF2BP2 SUMOylation in regulating VM and the function of IGF2BP2 in glioma cells.

The SUMO-site mutates (IGF2BP2-3KR) and knockdown IGF2BP2 cells were constructed. In comparison to the IGF2BP2-Wt group, cell proliferation, migration, invasion and VM of IGF2BP2-3KR group were significantly decreased and the expression of VM related proteins HIF1A and MMP14 also inhibited. Similar to above results, in comparison to the IGF2BP2(-)NC group, cell proliferation, migration, invasion and VM of IGF2BP2(-) group were significantly decreased and the expression of VM related proteins HIF1A and MMP14 also inhibited (Fig. 3C-F).

**Figure 4:**
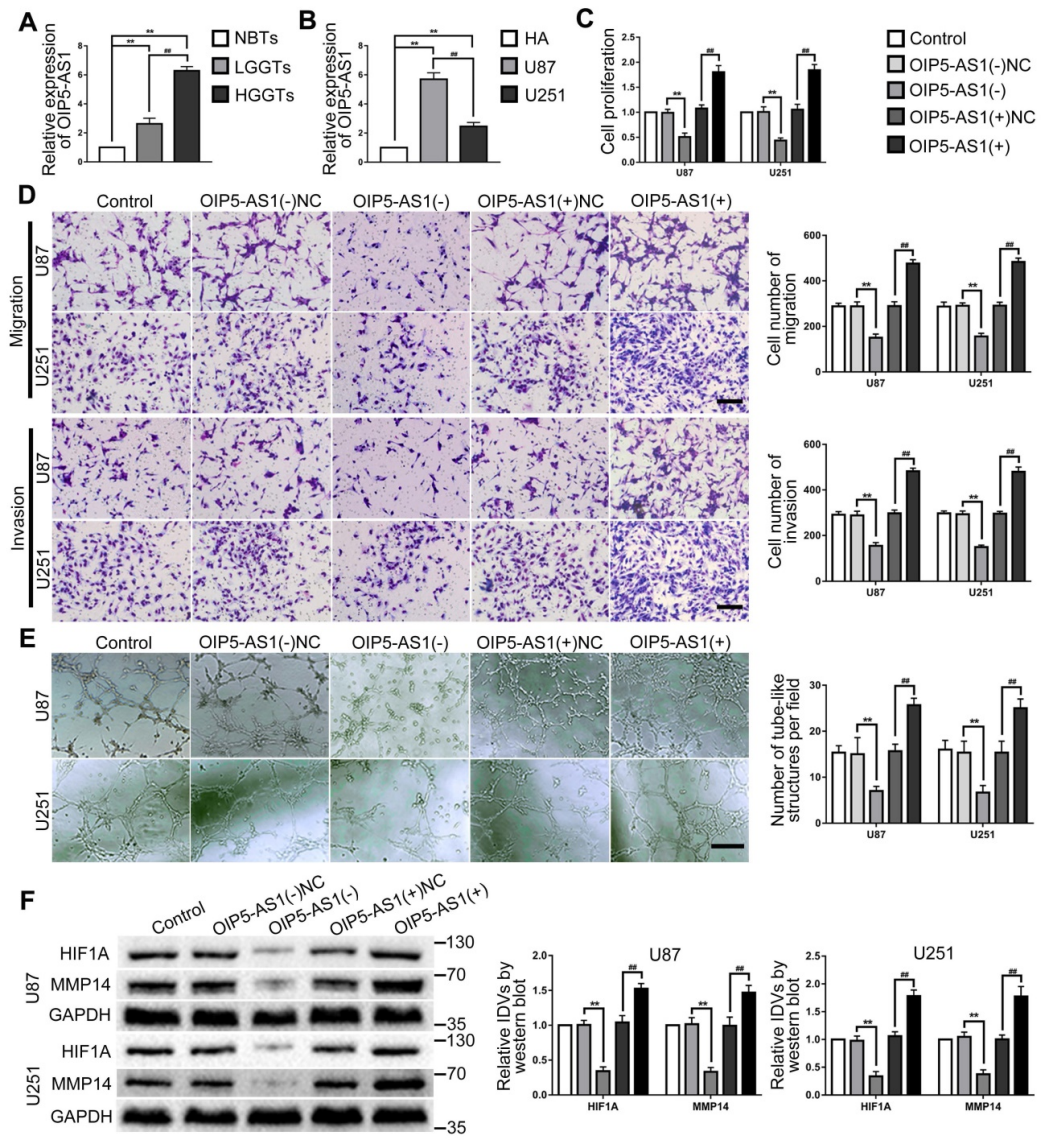
** OIP5-AS1 exerted tumor-promoting functions in glioma cells. (A)** RT-PCR was used to detected the expression levels of OIP5-AS1 in NBTs, LGGTs and HGGTs. Data are presented as the mean ± SD (n=3), **P<0.01, vs. NBTs, ##P<0.01, vs. LGGTs. **(B)** RT-PCR was used to detected the expression levels of OIP5-AS1 in HA cells, U87 cells and U251 cells. Data are presented as the mean ± SD (n=3), **P<0.01, vs. HA cells, ##P<0.01, vs. U87 cells. **(C-E)** CCK-8 assay, cell transwell assay, and three-dimensional cell culture method were used to detect the effects of knockdown and over-expression of OIP5-AS1 on proliferation, migration, invasion and VM formation. Data are presented as the mean ± SD (n=3), **P<0.01, vs. OIP5-AS1(-)NC, ##P<0.01, vs. OIP5-AS1(+)NC. Scale bar=50μm. **(F)** Western blot was used to detected the protein expression levels of HIF1A and MMP14 in glioma cells (U87 and U251) after knockdown and over-expression of OIP5-AS1. Data are presented as the mean ± SD (n=3), **P<0.01, vs. OIP5-AS1(-)NC, ##P<0.01, vs. OIP5-AS1(+)NC.

OIP5-AS1 expression in glioma tissues and cells were detected by RT-PCR. As the results showed, OIP5-AS1 was overexpressed in glioma tissues than in NBTs, and the expression increased with the pathological grade level. While the expression of OIP5-AS1 in U87 and U251 were significantly higher than that in HA cells (Fig. 4A-B). To further analyze the function of OIP5-AS1 in glioma cells, knockdown and overexpression plasmids of OIP5-AS1 were transfected into U87 and U251 cells. In comparison to the OIP5-AS1(-)NC group, cell proliferation, migration, invasion and VM of OIP5-AS1(-) group were significantly decreased and the expression of VM related proteins HIF1A and MMP14 also inhibited. On the contrary, cell proliferation, migration, invasion and VM of the OIP5-AS1(+) group were increased and the expression of VM related proteins HIF1A and MMP14 was also increased, by comparison to the OIP5-AS1(+)NC group (Fig. 4C-F).

### IGF2BP2 promoted VM in glioma cells by stabilizing and upregulating OIP5-AS1

**Figure 5:**
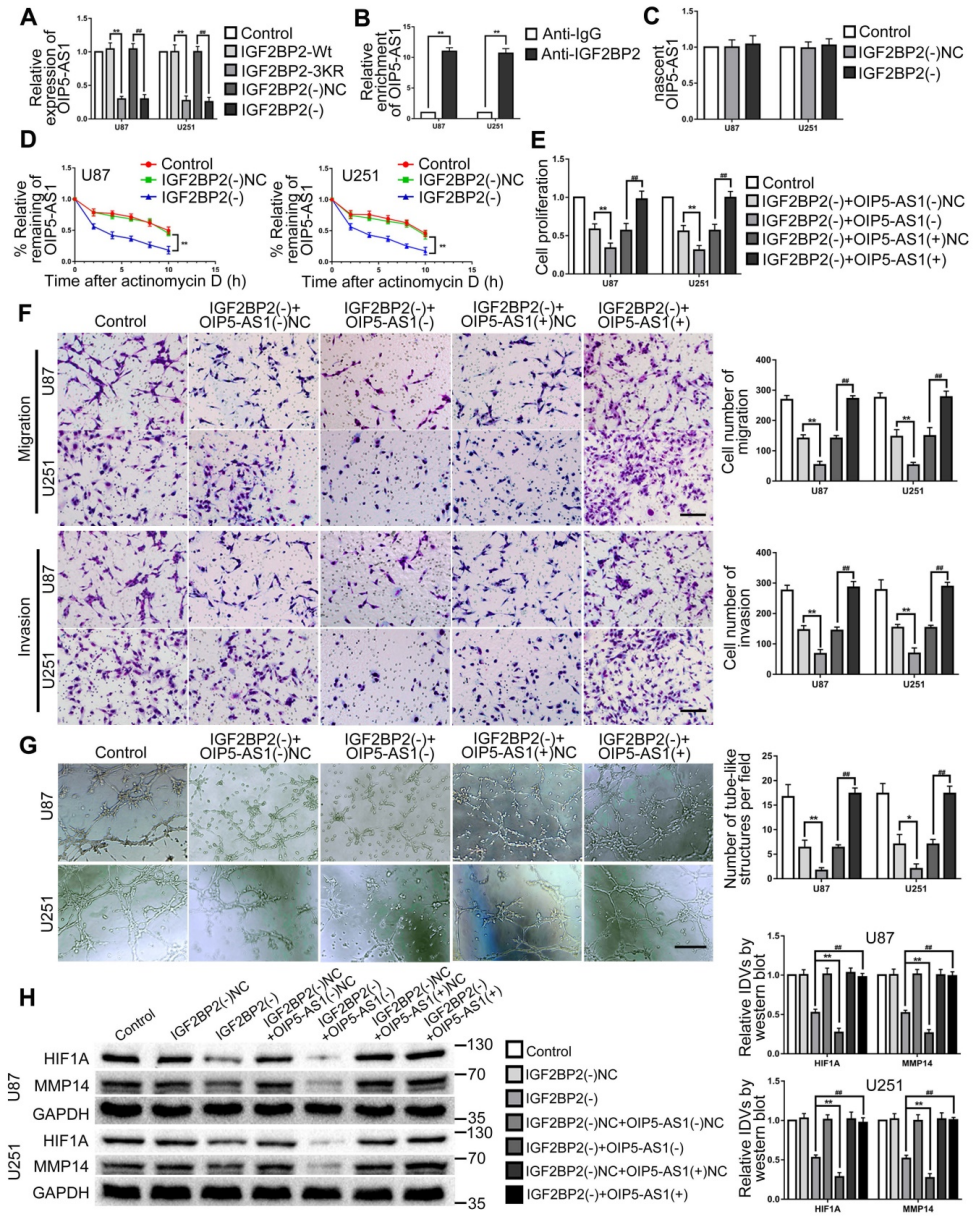
** IGF2BP2 enhanced the biological behavior of glioma cells by Increasing the Stability of OIP5-AS1. (A)** IGF2BP2 SUMOylation-sites mutated and IGF2BP2 knockdown reduce the expression of OIP5-AS1. Data are expressed as the mean ± SD (n=3). **p<0.01, vs. IGF2BP2-Wt. ##p<0.01, vs. IGF2BP2(-)NC. **(B)** Verifying the binding interaction between IGF2BP2 and OIP5-AS1. Data are expressed as the mean ± SD (n=3). **p<0.01 vs. anti-IgG. **(C)** Knockdown of IGF2BP2 to detect nascent OIP5-AS1 in glioma cells (U87 and U251). Data are expressed as the mean ± SD (n=3). **(D)** The effect of IGF2BP2 knockdown on OIP5-AS1 half-life. Data are expressed as mean ± SD (n=3). **p<0.01, vs. IGF2BP2(-)NC. **(E-G)** CCK-8 assay, cell transwell assay, and three-dimensional cell culture method were used to detect the effects of knockdown of IGF2BP2, knockdown and overexpression of OIP5-AS1 on proliferation, migration, invasion and VM formation. *p<0.05, **p<0.01, vs. IGF2BP2(-)+OIP5-AS1(-)NC. ##p<0.01, vs. IGF2BP2(-)+OIP5-AS1(+)NC. Scale bar=50μm. **(H)** Western blot was used to detected the protein expression levels of HIF1A and MMP14 in glioma cells (U87 and U251) after knockdown of IGF2BP2, knockdown and overexpression of OIP5-AS1. Data are presented as the mean ± SD (n=3), **p<0.01, ##p<0.01, vs. IGF2BP2(-).

This study further explored the effect of IGF2BP2 SUMOylated and IGF2BP2 knockdown on OIP5-AS1. The results revealed that OIP5-AS1 expression was significantly decreased by IGF2BP2 SUMOylation-sites mutated and knockdown (Fig. 5A). In order to definite the relationship between IGF2BP2 and OIP5-AS1, RNA binding protein immunoprecipitation (RIP) assay was used to detect the direct binding between IGF2BP2 and OIP5-AS1. The results showed that OIP5-AS1 was significantly enriched in the anti-IGF2BP2 group by comparison to the anti-IgG group (Fig. 5B). Then, the regulation mechanism of IGF2BP2 knockdown on OIP5-AS1 was further elucidated by nascent RNA capture and mRNA stability assay. The results showed that IGF2BP2 knockdown did not affect nascent OIP5-AS1 but significantly reduced the half-life of OIP5-AS1 (Fig. 5C-D). Knockdown of IGF2BP2 and knockdown or overexpression of OIP5-AS1 plasmid co-transfection in U87 and U251 cells to further detect their functions in glioma. The results revealed that cell proliferation, migration, invasion, and VM of IGF2BP2(-)+OIP5-AS1(-) group were significantly decreased when compared with the IGF2BP2(-)+OIP5-AS1(-)-NC group. In comparison with the IGF2BP2(-)+OIP5-AS1(+)NC group, in IGF2BP2(-)+OIP5-AS1(+) group, OIP5-AS1(+) reversed the inhibitory effect of IGF2BP2(-) on cell proliferation, migration, invasion, VM ability of glioma cells (Fig. 5E-G). Meantime, IGF2BP2 knockdown significantly inhibited HIF1A and MMP14 expression. Co-transfection of IGF2BP2 knockdown and OIP5-AS1 knockdown enhanced the inhibition effect caused by IGF2BP2 knockdown alone, while co-transfection of IGF2BP2 knockdown and OIP5-AS1 overexpression rescued the suppression effect (Fig. 5H).

### MiR-495-3p were lowly expressed in both glioma tissues and cells, miR-495-3p agomir inhibited VM of glioma cells

**Figure 6:**
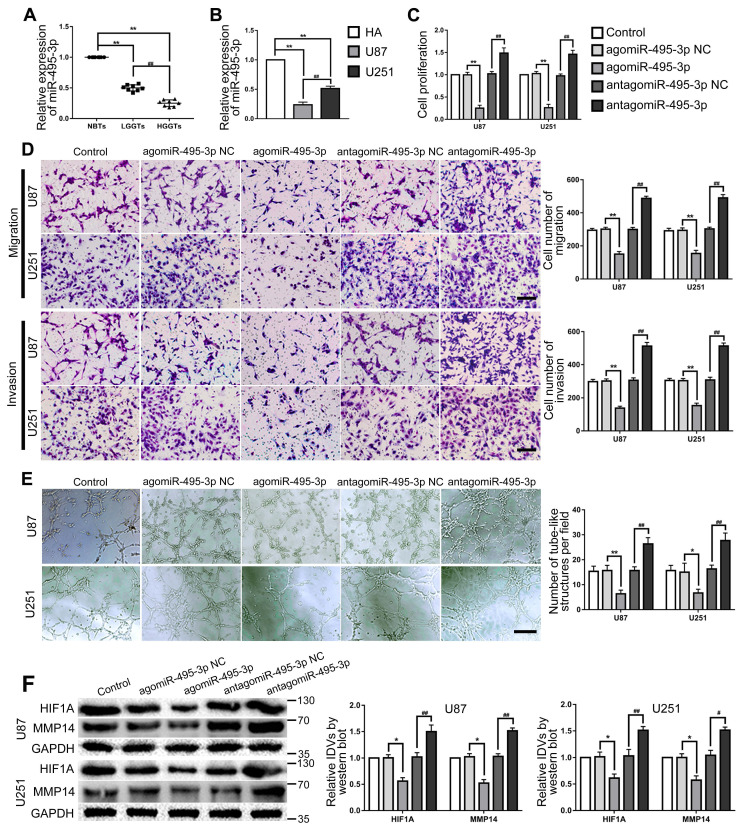
** MiR-495-3p exerted tumor-suppressing functions in glioma cells. (A)** RT-PCR was used to detected the expression levels of miR-495-3p in NBTs, LGGTs and HGGTs. Data are presented as the mean ± SD (n=8), **P<0.01, vs. NBTs, ##P<0.01, vs. LGGTs. **(B)** RT-PCR was used to detected the expression levels of miR-495-3p in HA cells, U87 cells and U251 cells. Data are presented as the mean ± SD (n=3), **P<0.01, vs. HA cells, ##P<0.01, vs. U87 cells. **(C-E)** CCK-8 assay, cell transwell assay, and three-dimensional cell culture method were used to detect the effects of agomiR-495-3p and antagomiR-495-3p on proliferation, migration, invasion and VM formation. Data are presented as the mean ± SD (n=3), *P<0.05, **P<0.01, vs. agomiR-495-3p NC, ##P<0.01, vs. antagomiR-495-3p NC. Scale bar=50μm. **(F)** Western blot was used to detected the effects of agomiR-495-3p and antagomiR-495-3p on the protein expression levels of HIF1A and MMP14 in glioma cells (U87 and U251). Data are presented as the mean ± SD (n=3), *P<0.05, vs. agomiR-495-3p NC, #P<0.05, ##P<0.01, vs. antagomiR-495-3p NC.

RT-PCR was used to detected miR-495-3p expression in glioma tissues and cells. The results confirmed that miR-495-3p expression was significantly down-regulated in glioma tissues and glioma cells (U87 and U251) (Fig. 6A-B). Up- or down-regulate miR-495-3p to further explore the function of miR-495-3p in glioma cells. Cell proliferation, migration, invasion and VM of the agomiR-495-3p group were significantly decreased, while the expression of VM related proteins HIF1A and MMP14 was inhibited. On the contrary, cell proliferation, migration, invasion and VM of antagomiR-495-3p group were significantly increased, meanwhile, the expression of VM related proteins HIF1A and MMP14 was increased (Fig. 6C-F).

### OIP5-AS1 targeted and negatively regulated miR-495-3p. Knockdown of OIP5-AS1 and overexpression of miR-495-3p inhibited VM of glioma cells

**Figure 7:**
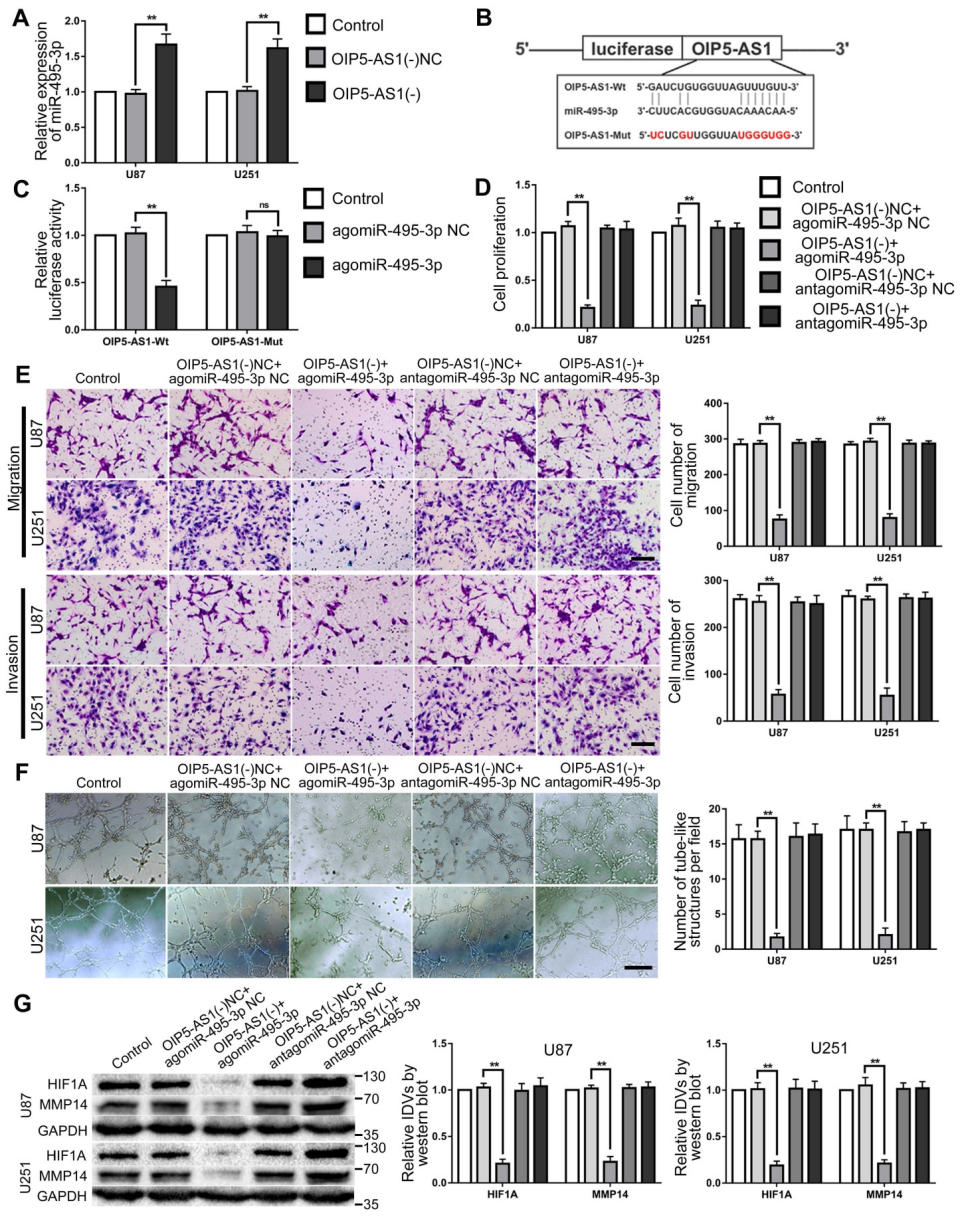
** MiR-495-3p target OIP5-AS1 and mediate the inhibitory effects of OIP5-AS1 knockdown on glioma cells. (A)** RT-PCR was used to detected the expression of miR-495-3p after OIP5-AS1 knockdown. Data are presented as the mean ± SD (n=3), **P<0.01, vs. OIP5-AS1(-) NC. **(B-C)** Predicted the binding sites of miR-495-3p in OIP5-AS1-Wt sequence and the designed mutant sequence of miR-495-3p binding site (OIP5-AS1-Mut). Relative luciferase activity was detected after cells were transfected with OIP5-AS1-Wt or OIP5-AS1-Mut. Data are presented as the mean ± SD (n=3). **P<0.01 vs agomiR-495-3p NC. **(D-F)** CCK-8 assay, cell transwell assay, and three-dimensional cell culture method were used to detect the effects of OIP5-AS1 and miR-495-3p on proliferation, migration, invasion and VM formation. Data are presented as the mean ± SD (n=3), **P<0.01, vs. OIP5-AS1(-)NC+agomiR-495-3p NC. Scale bar=50μm.**(G)** Western blot was used to detected the effects of OIP5-AS1 and miR-495-3p on the protein expression levels of HIF1A and MMP14 in glioma cells (U87 and U251). Data are presented as the mean ± SD (n=3), **P<0.01, vs. OIP5-AS1(-)NC+agomiR-495-3p NC.

RT-PCR was used to detect miR-495-3p expression in glioma cells after OIP5-AS1 knocked-down. The results confirmed that miR-495-3p expression level was significantly up-regulated in OIP5-AS1(-) group (Fig. 7A). The potential binding site between OIP5-AS1 and miR-495-3p was predicted by the bioinformatics database (Starbase 2.0). Dual-luciferase reporter gene assays results indicated that the relative luciferase activity of OIP5-AS1-Wt in the agomiR-495-3p group was significantly lower than in the agomiR-495-3p NC group, while in the agomiR-495-3p NC or agomiR-495-3p groups, the relative luciferase activity of OIP5-AS1-Mut did not significantly change (Fig. 7B-C). These results indicate that OIP5-AS1 targets miR-495-3p. To figure out if miR-495-3p affected the tumor suppressor function of glioma cells induced by OIP5-AS1 knockdown, U87 and U251 cells co-transfected with OIP5-AS1(-) and agomiR-495-3p or antagomiR-495-3p. The results indicated that in comparison to OIP5-AS1(-)NC + agomir-495-3p NC group, cell proliferation, migration, invasion and VM of OIP5-AS1(-)+agomir-495-3p group were significantly decreased. The expression of VM-associated proteins HIF1A and MMP14 were significantly reduced (Fig. 7D-G).

### MiR-495-3p inhibited the expression of HIF1A and MMP14 by directly binding their 3'UTR, thus inhibiting the VM of glioma cells

**Figure 8:**
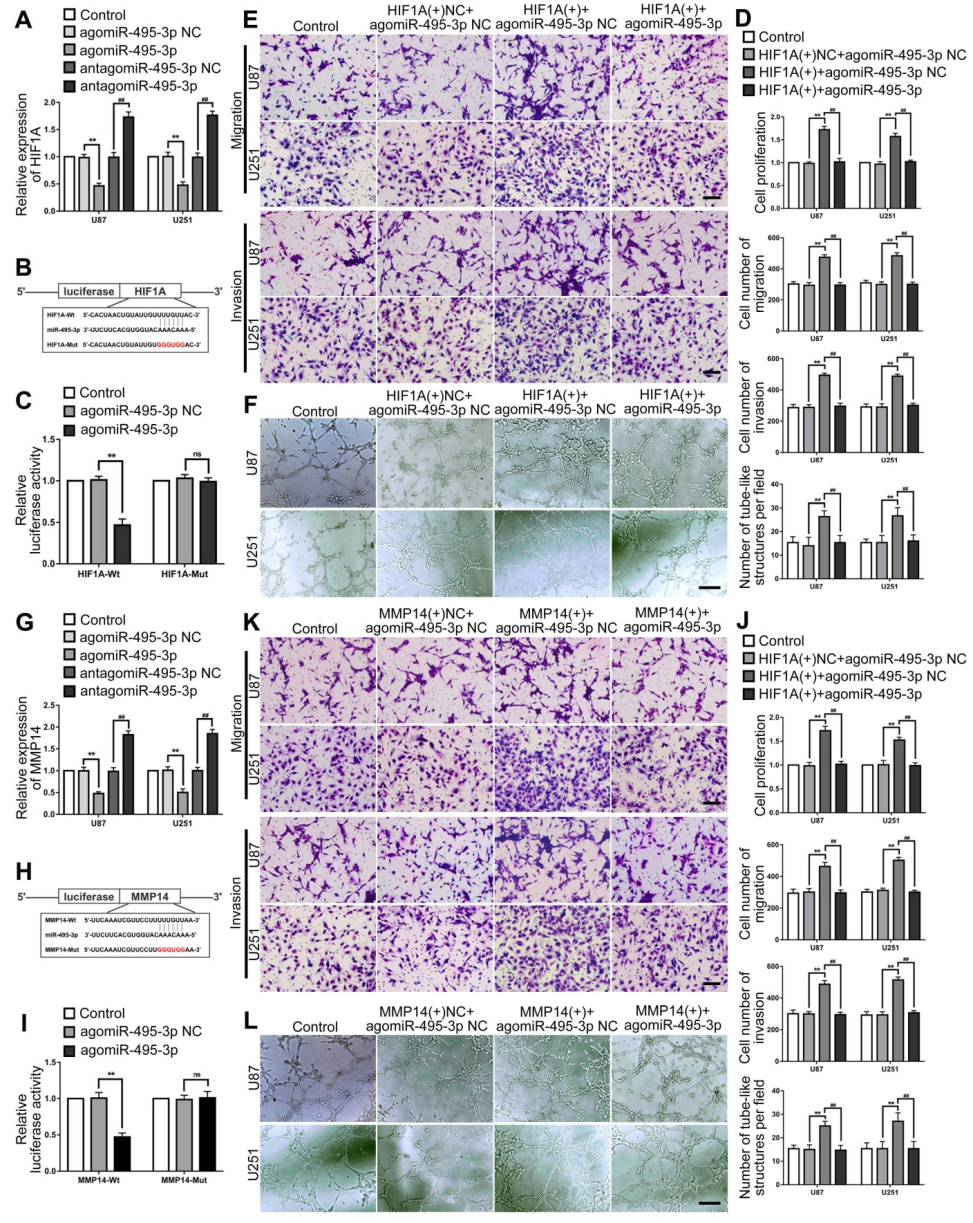
** HIF1A and MMP14 were the target of miR-495-3p, could mediated and rescue the tumor-suppressive effects of miR-495-3p. (A)** RT-PCR was used to detected the expression of HIF1A after miR-495-3p agomir or antagomir. Data are presented as the mean ± SD (n=3), **P<0.01, vs. agomiR-495-3p NC, ##P<0.01, vs. antagomiR-495-3p NC. **(B-C)** Predicted the binding sites of miR-495-3p in HIF1A-Wt sequence and the designed mutant sequence of miR-495-3p binding site (HIF1A-Mut). Relative luciferase activity was detected after cells were transfected with HIF1A-Wt or HIF1A-Mut. Data are presented as the mean ± SD (n=3). **P<0.01 vs agomiR-495-3p NC. **(D-F)** CCK-8 assay, cell transwell assay, and three-dimensional cell culture method were used to detect the effects of miR-495-3p and HIF1A on proliferation, migration, invasion and VM formation. Data are presented as the mean ± SD (n=3), **P<0.01, vs. HIF1A(+)NC+agomiR-495-3p NC. ##P<0.01, vs. HIF1A(+)+agomiR-495-3p NC. Scale bar=50μm. **(G)** RT-PCR was used to detected the expression of MMP14 after miR-495-3p agomir or antagomir. Data are presented as the mean ± SD (n=3), **P<0.01, vs. agomir-495-3p-NC, ##P<0.01, vs. antagomir-495-3p-NC. **(H-I)** Predicted the binding sites of miR-495-3p in MMP14-Wt sequence and the designed mutant sequence of miR-495-3p binding site (MMP14-Mut). Relative luciferase activity was detected after cells were transfected with MMP14-Wt or MMP14-Mut. Data are presented as the mean ± SD (n=3). **P<0.01 vs agomiR-495-3p NC. **(J-L)** CCK-8 assay, cell transwell assay, and three-dimensional cell culture method were used to detect the effects of miR-495-3p and MMP14 on proliferation, migration, invasion and VM formation. Data are presented as the mean ± SD (n=3), **P<0.01, vs. MMP14(+)NC+agomiR-495-3p NC. ##P<0.01, vs. MMP14(+)+agomiR-495-3p NC. Scale bar=50μm.

Up- or down-regulated miR-495-3p expression level in glioma cells to evaluated the the mRNA expression of HIF1A by RT-PCR. The results indicated that HIF1A expression was significantly decreased after miR-495-3p agomir, while was significantly increased after miR-495-3p antagomir (Fig. 8A). The HIF1A 3'UTR existed a direct binding site of miR-495-3p which was predicted by the bioinformatics database (Starbase 2.0). Dual-luciferase reporter gene assays results revealed that the relative luciferase activity of HIF1A-Wt in the agomiR-495-3p group was significantly lower than in the agomiR-495-3p NC group, while in the agomiR-495-3p NC or agomiR-495-3p groups, the relative luciferase activity of OIP5-AS1-Mut did not significantly change (Fig. 8B-C). To figure out if HIF1A could reverse the tumor suppression effects of miR-495-3p on U87 and U251 cells, HIF1A(+) and agomiR-495-3p were transfected into cells and evaluated the biological behaviors. We found that the HIF1A overexpression significantly increased cell proliferation, migration, invasion and VM of glioma cells, and reverse the tumor-suppressive effect of miR-495-3p up-regulated (Fig. 8D-F). Similarly, similar results were observed in detecting the interaction between MMP14 and miR-495-3p (Fig. 8G-L).

### The inhibition of SUMOylation of IGF2BP2 could inhibit tumor xenograft growth, prolong the survival time and yield the lowest VM formation of nude mice

**Figure 9:**
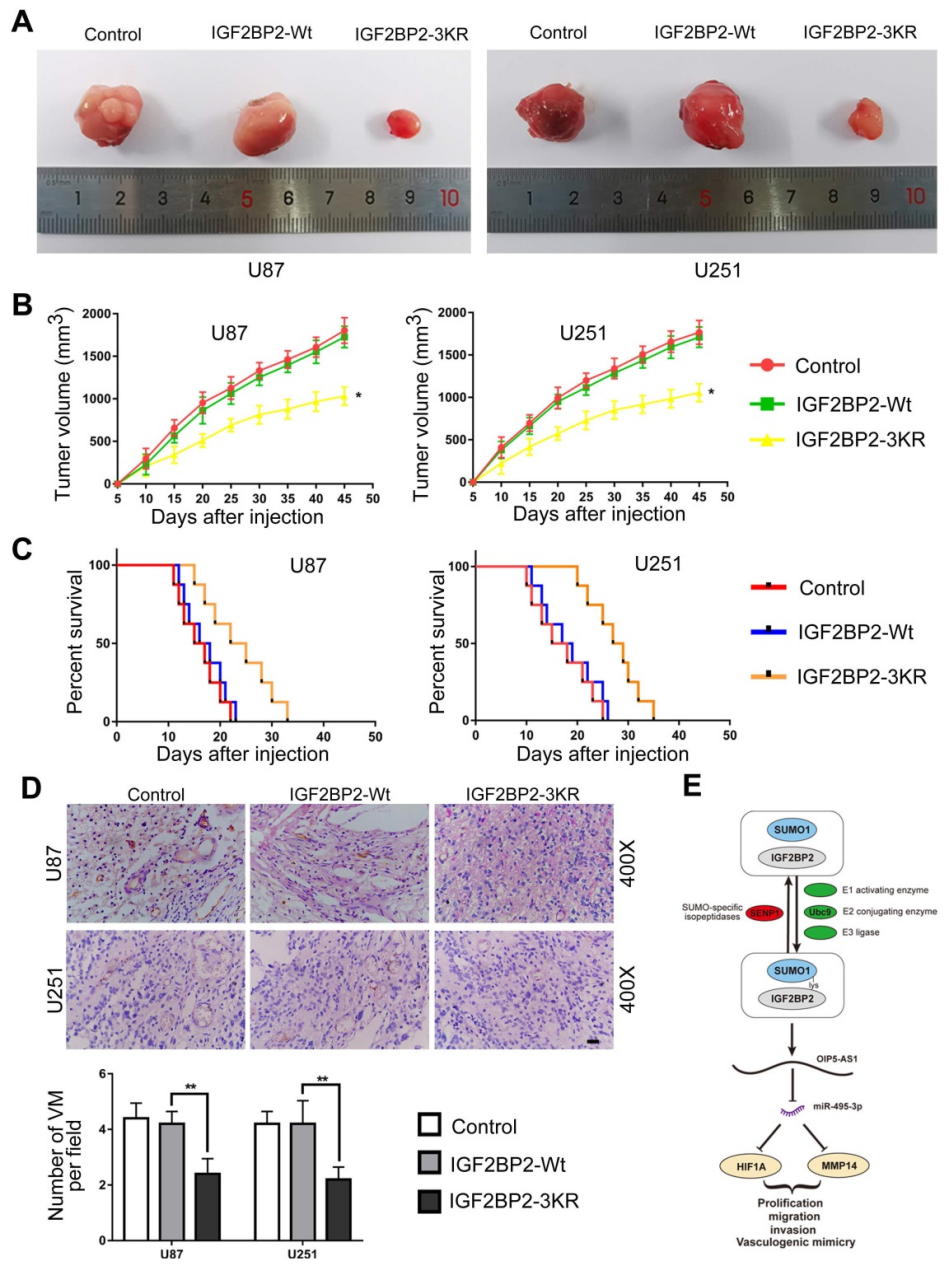
** In vivo detection of IGF2BP2 SUMOylation effects of migration, invasion, and VM on glioma cells. (A)** Sample tumors from each group of subcutaneous xenograft tumors. **(B)** Tumor growth curves. Tumor volume was calculated every 5 days after injection, and tumors were taken 45 days after injection. Data are presented as the mean ± SD (n=3). *p<0.05, vs. IGF2BP2-Wt. **(C)** Survival curves from representative nude mice injected into the right striatum (n=8, each group). **(D)** CD34-PAS staining was used to detect the effects of IGF2BP2 SUMOylation on VM in xenograft tumors tissue. Data are presented as the mean ± SD (n=3). **p<0.01, vs. IGF2BP2-Wt. Scale bar=100μm. **(E)**The schematic diagram underlying the mechanism that IGF2BP2 SUMOylation promotes vasculogenic mimicry of glioma via regulating OIP5-AS1/miR-495-3p axis.

The function of IGF2BP2 SUMOylation on glioma cells was further determined by using the subcutaneous and orthotopic transplantation model. As the results presented, in comparison to IGF2BP2-Wt group, the tumor volumes were significantly lower in IGF2BP2-3KR group (Fig. 9A-B). In addition, survival analysis showed that nude mice in IGF2BP2-3KR group have a longer survival time than in the IGF2BP2-Wt group (Fig. 9C). Last, the CD34-PAS dual-staining was used to detect the VM in different groups. The VM density in IGF2BP2-3KR group was lower than that in IGF2BP2-Wt group (Fig. 9D).

## Discussion

SUMO protein, as the starting molecule of SUMOylation, initiates the process of SUMOylation by covalently binding with lysine residues of substrate protein. Numbers of studies have shown that SUMOylation can influence the stability, subcellular localization and protein activity of substrate protein, and then have a key impact on the development of tumor. For example, in non-small cell lung cancer, METTL3 combines with SUMO1 to make SUMO modification of METTL3 regulate its m6A RNA methyltransferase activity, resulting in the decrease of m6A level in mRNAs, and ultimately affect the growth of cell colonies and xenograft tumors 38. Slug protein interacts with Ubc9 and SUMO1, and realizes SUMO modification in cells. Furthermore, more HDAC1 was recruited to enhance the transcriptional inhibitory activity of Slug, resulting in a decrease in the expression of target genes downstream of Slug, thereby promoting the metastasis of non-small cell lung cancer 39. In this study, we first found that IGF2BP2 was mainly combined with SUMO1 in cells and SUMOylation occurred. Subsequently, we verified the role of key enzymes (Ubc9 and SENP1) in promoting or de-SUMOylation in this process. Based on the prediction and analysis of GPS-SUMO (www.sumosp.biocuckoo.org/online.php), SUMOplot (www.abcepta.com/sumoplot), and JASSA (http://www.jassa.fr/index.php?m=jassa), the results of Ni^2+^-NTA agarose bead pull-down assays showed that IGF2BP2 mainly combined with SUMO1 at K497, K505 and K509 sites, and SUMOylation of IGF2BP2 was inhibited more obviously when the three binding sites mutated together (3KR, K497/505/509R).

Numbers of studies had shown that SUMOylation plays an important role in regulating of substrate protein degradation 40, 41.To further clarify the effect of SUMOylation on IGF2BP2 expression, the study found that when the site mutation (3KR, K497/505/509R), the inhibited SUMO modification significantly reduced the translation level of IGF2BP2 protein but did not affect its mRNA transcription level. With the existence of CHX, the expression of IGF2BP2 increased which caused by SUMO1 overexpressed had indicated that its degradation was reduced, while SENP1 reversed the degradation process. However, the expression level of IGF2BP2-3KR was not significantly affected by SUMO1 and SENP1. The above results showed that SUMOylation increased the stability of IGF2BP2, but the inhibited SUMOylation after site mutation lost the enhancement effect on the stability of substrate protein. Further study on the degradation process of the substrate protein IGF2BP2 showed that the degradation speed of IGF2BP2-3KR was faster than that of IGF2BP2-Wt, and the degradation process could be blocked by MG132, rather than chloroquine, by further research we found that SUMOylation increases IGF2BP2 stability by antagonizing ubiquitination. This indicated that IGF2BP2 was mainly degraded by ubiquitin-proteasome pathway, and the inhibited SUMOylation after site mutation could accelerate the degradation of substrate protein. Nucleoplasmic separation and immunofluorescence results showed that IGF2BP2 was mainly distributed in the cytoplasm, the SUMOylation after site mutation did not affect the subcellular localization of IGF2BP2.

As a member of the IGF2 mRNA-binding protein family, IGF2BP2 acts a key role in a wide range of pathological and physiological functions such as embryonic development, neuronal differentiation, energy metabolism and tumorigenesis 42-45. Recent studies have shown that IGF2BP2 acts as an oncogene in a variety of tumors. For example, high expression of IGF2BP2 in colon cancer cells, inhibited the expression of IGF2BP2 significantly inhibited the growth, invasion and migration of LoVo cells, and reduced the weight and microvessel density of tumor 46. IGF2BP2 also over-expressed in non-small cell lung cancer. Inhibition of IGF2BP2 expression significantly reduces cell proliferation and invasion of NSCLC cells, and IGF2BP2 overexpressed reverses the anti-tumor effects of miR-485-5p in tumor cells^47^. Recent studies also confirm that IGF2BP2 participates in the regulation of tumor cell proliferation by regulating signaling pathways in tumor development and development. For example, up-regulating IGF2BP2 expression can promote pancreatic cancer cell proliferation through activating PI3K/Akt signaling pathway 23. IGF2BP2 mediates the HBX-miR-216b-IGF2BP2 signaling pathway and regulates the progression of hepatocellular carcinoma 48. The reports above fully confirm that IGF2BP2 plays an essential role during the complicated tumor progression process. In this study, western blot was found the high expression of IGF2BP2 in glioma tissues and cells, and IGF2BP2 expression level was positively correlated with pathological grade of glioma. IGF2BP2 knockdown significantly reduced cell proliferation, migration, invasion and VM formation of glioma cells. Meanwhile, SUMOylation of IGF2BP2 inhibited by site mutation significantly inhibited cell proliferation, migration, invasion and VM ability of glioma cells. The above results showed that IGF2BP2 may play as an oncogene in glioma, SUMO modification of IGF2BP2 promotes the formation of VM in glioma.

As a long-chain non-coding RNA, OIP5-AS1 plays an essential regulatory role in the development of tumors, and its regulatory role in cell proliferation, migration, invasion and other cellular functions has been confirmed. OIP5-AS1 is overexpressed in ovarian cancer, and overexpression of OIP5-AS1 as a molecular sponge adsorbs miR-34a to promote tumor cell migration and invasion 49. The expression of OIP5-AS1 was increased in pancreatic ductal adenocarcinoma. Overexpression of OIP5-AS1 increased FOXD1 expression and activated ERK pathway by targeting miR-429, which played a carcinogenic role in PDAC cells 50. In this study, we found that OIP5-AS1 was over-expressed in both glioma tissues and cells. Knockdown of OIP5-AS1 significantly inhibited cell proliferation, migration, invasion and VM formation of glioma cells.

More and more studies have revealed that RBPs and lncRNAs binding and interacting with each other in a variety of tumors, RBPs up-regulate lncRNA expression by enhancing its stability, and then regulate the occurrence and development of tumors 51, 52. For example, A1CF targets binding and increases the stability of FAM224A, thereby increasing the expression of FAM224A, then reducing the malignant biological behavior of glioma 53. In this study, both SUMOylation-sites mutated and knockdown of IGF2BP2 can reduce the expression of OIP5-AS1. The RIP experiment verified the direct binding between IGF2BP2 and OIP5-AS1. Knockdown of IGF2BP2 did not affect the nascent OIP5-AS1, but significantly reduced the half-life of OIP5-AS1, indicating IGF2BP2 could up-regulate OIP5-AS1 expression level by enhancing its stability. Knockdown of IGF2BP2 and OIP5-AS1 significantly inhibited cell proliferation, migration, invasion and VM formation of glioma cells. OIP5-AS1 overexpression rescued the tumor suppressor effect after IGF2BP2 knockdown. The above results showed that IGF2BP2 could bind to OIP5-AS1 and promote the stability of OIP5-AS1 in glioma cells, thereby promoting the formation of glioma VM.

This study further confirmed that miR-495-3p expression was down-regulated in glioma tissues and cells, and its expression level decreased with the pathological grade increasing. Up-regulated of miR-495-3p expression can significantly inhibit cell proliferation, migration, invasion and VM formation of glioma cells, which indicated that miR-495-3p acted as a tumor suppressor gene in glioma. Consistent with our results, miR-495-3p also acts as an anti-tumor gene in ovarian cancer and interacts with LINC01133 to promote epithelial ovarian cancer metastasis by regulating TPD52 54. MiR-495-3p is low-expressed in gastric cancer, and SNHG10 promotes cell proliferation and migration of gastric cancer cells by targeting miR-495-3p regulating CTNNB1 55.

LncRNA can regulate miRNAs through competitive endogenous RNA (ceRNA) mechanism and inhibit the regulation of miRNAs on downstream target genes, so it is called “miRNA sponge” 56, 57. For example, RP11-619L19.2 regulates the expression of CD164 and EMT through sponge adsorption of miR-1271-5p, and promotes the development of colon cancer 58. ADAMTS9-AS1 inhibits the invasion of breast carcinoma cells by regulating ZFP36 via sponging miR-513a-5p 59. In this study, we first found that knockdown of OIP5-AS1 could significantly up-regulate the expression of miR-495-3p. For further understanding the potential regulatory mechanism between OIP5-AS1 and miR-495-3p, we predicted the binding between OIP5-AS1 and miR-495-3p based on the database (starBase2.0) and Dual-luciferase reporter gene assays. Knockdown of OIP5-AS1 and overexpression of miR-495-3p can significantly inhibit cell proliferation, migration, invasion and VM capability of glioma cells. The above results show that inhibiting the expression of OIP5-AS1 can up-regulate the expression of miR-495-3p through competitive endogenous RNA mechanism, thereby inhibiting the proliferation, migration, invasion and VM capability of glioma cells.

Studies have shown that HIF1A and MMP14, as VM-related effector proteins, play the roles of oncogenes in a variety of tumors 11,35-37. This study first found that knockdown or overexpression of miR-495-3p can significantly change HIF1A expression level in glioma cells. In order to further understand the potential regulatory mechanism between miR-495-3p and HIF1A, we predicted the binding between miR-495-3p and HIF1A based on the starBase2.0 database and Dual-luciferase reporter gene assays. Overexpression of HIF1A can rescued the inhibitory effect of miR-495-3p on the proliferation, migration, invasion and VM formation ability of glioma cells. Similar results were also observed in the verification of the regulatory mechanism between miR-495-3p and MMP14. The above results show that miR-495-3p can target binding and regulate VM-related effector protein HIF1A and MMP14, thereby regulating the formation of VM in glioma.

Finally, the effect of SUMOylation of IGF2BP2 on the growth of xenografts in nude mice was verified in vivo. In comparison with IGF2BP2-Wt group, transplanted tumor size in IGF2BP2-3KR group was significantly reduced, whereas survival time of nude mice was significantly prolonged. The above results show that inhibit the SUMOylation of IGF2BP2 reduce glioma tumor growth, also means IGF2BP2 SUMOylation has potential therapeutic value.

In summary, this study revealed for the first time that SUMOylation of IGF2BP2 promotes vasculogenic mimicry of glioma via regulating OIP5-AS1/miR-495-3p axis. We found that the expression of IGF2BP2 and OIP5-AS1 were up-regulated in glioma tissues and cells, while miR-495-3p was down-regulated. The SUMOylation of IGF2BP2 was mainly induced by the binding between IGF2BP2 and SUMO1. SUMO-site mutant (IGF2BP2-3KR) inhibited IGF2BP2 SUMOylation, resulting in accelerated degradation and decreased stability of IGF2BP2 and decreased the expression level of IGF2BP2 protein. In addition, knockdown of IGF2BP2 reduced the stability and expression level of OIP5-AS1, thereby reducing the combination of OIP5-AS1 and miR-495-3p, enhancing the negative regulation of miR-495-3p on HIF1A and MMP14, and ultimately inhibiting the proliferation, migration, invasion and VM in glioma cells. The results of this study confirmed an important mechanism of IGF2BP2 SUMOylation in regulating VM of glioma. IGF2BP2, OIP5-AS1 and miR-495-3p may become new targets for molecular targeted therapy of glioma, and the research on the treatment of gliomas needs to be further studied.

## Materials and methods

### Pathological tissue specimens and cell culture

The human glioma tissues and normal brain tissues involved in this experiment were obtained from the Department of Neurosurgery of Shengjing Hospital of China Medical University. All patients signed the informed consent form voluntarily. This research has already approved by the ethics committee of Shengjing Hospital of China Medical University. The cells (HEK293T, U87, U251 and HA cells) involved in this experiment were purchased from the biotechnology company (Shanghai Gene Chemistry Co., Ltd, and Shanghai Zeye Biotechnology). More details of storage and cell culture, please refer to online Additional Materials and Methods.

### Reverse transcription and qRT-PCR

Quantitative real-time PCR (qRT-PCR) was used to determine the RNA expression of each indicator in this study. The RNA of tissues and cells were extracted by Trizol reagent (Life Technologies Corporation, Carlsbad, CA, USA). The RNA expression of each indicator in this study was quantitatively analyzed by The 7500 Fast RTPCR System. More details refer to the Additional Materials and Methods.

### Western blot

Western blot was used to detect the protein expression of each indicator in this study. More details refer to the Additional Materials and Methods.

### Cell transfection

The indicate plasmids (overexpression, knockdown, or site-mutant) of each indicator was used to transfected different cells. Stable transfected and resistant cell clones were screened and established by G418, and Puromycin. More details refer to the Additional Materials and Methods.

### RNA binding protein immunoprecipitation assays

The bounding between IGF2BP2 and OIP5-AS1 were detected by RNA binding protein immunoprecipitation (RIP) assays. More details refer to the Additional Materials and Methods.

### Ni^2+^-NTA agarose beads pull-down assay

SUMOylation of IGF2BP2 was analyzed in cells by using SUMOylation assay with Ni^2+^-NTA agarose beads. More details refer to the Additional Materials and Methods.

### Immunoprecipitation (IP)

The binding and modification of endogenous SUMO1 on endogenous IGF2BP2 was detected by CO-IP. The effect of IGF2BP2 SUMOyalted on ubiquitination was detected by IP. More details refer to the Additional Materials and Methods.

### Cell Counting Kit-8 (CCK-8) assay

CCK-8 assays were used to evaluate the cell proliferation level in this study. More details refer to the Additional Materials and Methods.

### Cell Transwell method

The ability for migration and invasion of glioma cells was detected by transwell method in vitro. More details refer to the Additional Materials and Methods.

### Reporter vectors construction and luciferase reporter assays

The responsive miR-495-3p binding sites in the OIP5-AS1, HIF1A 3'UTR and MMP14 3'UTR were determined by dual-luciferase reporter system. More vectors construction and experiment details refer to the Additional Materials and Methods.

### Tumor xenograft implantation in nude mice

The stably transfected cells were constructed with indicated plasmids, and then xenografted into immunodeficient nude mice for in vivo experiment in this study. More details refer to the Additional Materials and Methods.

### Immunofluorescence staining

The subcellular localization and expression of IGF2BP2 SUMOylated was detected by immunofluorescence staining in this study. More details refer to the Additional Materials and Methods.

### Cells VM formation assays

Three-dimensional cell culture method was used to detect VM formation ability of glioma cells in vitro, Matrigel Basement Membrane Matrix (BD Biosciences, Bedford, MA, USA) was treated as medium in this study. More details refer to the Additional Materials and Methods.

### CD34 endothelial marker periodic Acid-Schiff dual staining (CD34-PAS)

The qualitative and quantitative analyses of VM in tumor xenograft tissue sections of nude mice were detected by CD34-PAS assays. More details refer to the Additional Materials and Methods.

### Statistical analysis

Data of this study were expressed as the mean ± standard deviation (SD) and were analyzed by GraphPad Prism 7.0 statistical software with the t-test or one-way ANOVA.

## Supplementary Material

Supplementary materials and methods, figure.

## Figures and Tables

**Table 1:** Primer sequences for qRT-PCR

Target gene	Primer sequences
**IGF2BP2**	Forward 5'-GATGAACAAGCTTTACATCGGG-3'
	Reverse 5'-GATTTTCCCATGCAATTCCACT-3'
**OIP5-AS1**	Forward 5'-TGCAACCCAAGGTGGATACT-3'
	Reverse 5'-GAGAGACTGCAGTGAGCAGA-3'
**HIF1A**	Forward 5'-ACTGCACAGGCCACATTCACG-3'
	Reverse 5'-AATCAGCACCAAGCAGGTCATAGG-3'
**MMP14**	Forward 5'-CAAGATTGATGCTGCTCTCTTC-3'
	Reverse 5'-ACTTTGATGTTCTTGGGGTACT-3'
**GAPDH**	Forward 5'-GGAAGCTTGTCATCAATGGAAATC-3'
	Reverse 5'-TGATGACCCTTTTGGCTCCC-3'

## Abbreviations

SUMO: small molecule ubiquitin-like modifier
IGF2BP2: insulin-like growth factor 2 mRNA-binding protein 2
SENPs: SUMO-specific proteases
OIP5-AS1: OIP5 antisense RNA 1
HIF1A: hypoxia inducible factor 1 subunit alpha
ceRNA: competitive endogenous RNA
CHX: cycloheximide
CCK-8: cell counting kit-8
LncRNAs: long non-coding RNAs
MMP14: matrix metalloproteinase 14
NBTs: normal brain tissues
LGGTs: low grade glioma tissues
HGGTs: high grade glioma tissues
NHA: normal human astrocyte
PAS: periodic acid-schiff
qRT-PCR: quantitative real-time PCR
RBPs: RNA-binding proteins
VM: vasculogenic mimicry
