# Supplementary material for: SUMOylation of IGF2BP2 promotes vasculogenic mimicry of glioma via regulating OIP5-AS1/miR-495-3p axis
Source: Int J Biol Sci. 2021 Jul 13;17(11):2912–30. doi: 10.7150/ijbs.58035 (PMC8326132; doi:10.7150/ijbs.58035)
Supplement: Supplementary file 1 — Supplementary materials and methods, figure. [file ijbsv17p2912s1.pdf]

Additional Materials and Methods

Pathological tissue acquisition and cell culture

All human glioma tissues and normal brain tissues were obtained from patients at the Department of Neurosurgery of Shengjing Hospital of China Medical University. All samples were immediately frozen in liquid nitrogen after surgical and stored at -80°C before used. All patients voluntarily signed informed consent, and this study has been approved by the Ethics Committee of Shengjing Hospital of China Medical University. According to the 2007 WHO classification of tumors in the central nervous system, all glioma tissue samples were divided into low-grade glioma tissues group (WHO I-II) and high-grade glioma tissues group (WHO III-IV) by neuropathologists. Human astrocyte (HA) cells were purchased from Shanghai Zeye Biotechnology and grown in RPMI-1640 culture medium (Gibco, Grand Island, NY, USA) with 10% fetal bovine serum (FBS, Gibco, Carlsbad, CA, USA). Human glioma U87 and U251 cells and human embryonic kidney (HEK) 293T cells were purchased from Shanghai Genechem Co, Ltd, and grown in Dulbecco's modified Eagle medium (DMEM)/high glucose with 10% FBS. All cells were maintained in a humidified incubator at 37°C with 5% CO2.

Reverse transcription and quantitative real-time PCR (qRT-PCR)

Trizol reagent (Life Technologies Corporation, Carlsbad, CA) was used to extract total RNA (human tissue samples and HA, U87 and U251 cells). The ratio of 260/280 nm of Nanodrop Spectrophotometer (ND-100, Thermo, USA) was used to measured and determined the RNA concentration and quality. The 7500 Fast RT-PCR System (Applied Biosystem, USA) and One Step TB Green PrimeScript™ RT-PCR Kits (Perfect Real Time) (Takara Bio, Inc., Japan) were used to detect the expression of IGF2BP2, OIP5-AS1, HIF1A and MMP14. Simultaneously, GAPDH was regarded as the endogenous control. TaqMan MicroRNA Reverse Transcription kit and High Capacity cDNA Reverse Transcription Kit were used for miRNA reverse transcription. The primers for hsa-miR-495-3p and U6 were synthesized by the Thermofisher Scientific. Quantitative real-time PCR (qRT-PCR) was conducted using TaqMan Universal Master Mix II with TaqMan microRNA assays of miR-495-3p and U6 using the ABI 7500 Fast Real-Time PCR System (Applied Biosystems). Expressions were normalized to endogenous controls and fold change was determined using the formula of $2^{-\Delta\Delta Ct}$.

Western blot

The RIPA (Beyotime Institute of Biotechnology) buffer were used to lyse the harvested glioma cells (U87 and U251) on ice about 30 min and centrifuged at 4 ºC and 17,000g for 50 min. The BCA protein assay kit (Beyotime Institute of Biotechnology, Jiangsu, China) was used for the protein concentration measurement. Subjected the samples to SDS-PAGE electrophoretically, and then transferred to PVDF membranes. All membranes were blocked by Tween-Tris-buffered saline (TTBS) containing 5% non-fat milk for 2 h at room temperature and then incubated with primary antibodies as follows: anti-HA (1:500, from Abcam), anti-His (1:500, from Abcam), anti-Flag (1:500, from Abcam), anti-Myc (1:500, from Abcam), SUMO1 (1:500, from Santa Cruz), SENP1 (1:500, from CST), LaminB1 (1:500, from Proteintech), IGF2BP2 (1:500, from Proteintech), HIF1A(1:500, from Proteintech), MMP14 (1:500, from Proteintech) and GAPDH (1:5000, from Proteintech) overnight at 4 ºC. At room temperature, all the membranes were washed by TTBS three times, and then incubated with horseradish peroxidase II antibody (1:10000; from Proteintech) for

2 hours. The synergistic chemical imager (ECL) kit (Santa Cruz Biotechnology) was used for staining protein gels and then used the manufacturer's ChemImager 5500 V2.03 software scan. CHX chase assays was used to detect the steady-state levels of IGF2BP2. Briefly, after 24 hours of cell transfections, CHX was added to the cell culture, and all samples were prepared at indicated time, and then followed by Western blot with corresponding antibodies. Extraction of cytoplasmic and nuclear proteins was performed using the Nuclear and Cytoplasmic Protein Extraction Kit (Beyotime) according to its instruction, and then followed by Western blot with corresponding antibodies.

Cell transfection

HA-IGF2BP2 was obtained by using standard cloning procedures that amplified and subcloned the human IGF2BP2 cDNA into the vectors pCMV-HA. By using the QuikChange Lightning Site-Directed Mutagenesis Kits (Stratagene), the HA-IGF2BP2-mut (K497R, K505R, K509R, K497/505/509R) were generated via site-directed mutagenesis. His-SUMO1, His-SUMO2, His-SUMO3, Flag-Ubc9 and myc-SENP1 were purchased from GenePharama. Short hairpin RNA (shRNA) against Ubc9, SENP1, IGF2BP2, OIP5-AS1 gene, as well as their non-targeting sequences were constructed in pGPU6/GFP/Neo vector (GenePharama, Shanghai, China). Full-length OIP5-AS1, HIF1A or MMP14 gene were constructed in pIRES2-EGFP (GenScript, Piscataway, NJ, USA). MiR-495-3p agomir and miR-495-3p antagomir were synthesized (GenePharama, Shanghai, China). Plasmid carrying a non-targeting sequence was used as a negative control. When the cells confluence rate over 70%, Lipofectamine 3000 Reagents (Life Technologies, Carlsbad, CA, USA) were used for the cell transfection, and the stable transfected cells were selected by the G418 and puromcin (Sigma-Aldrich, StLouis, MO, USA). About 4 weeks, the resistant cell clones were established. Transfected efficiencies were detected by qRT-PCR and western blot.

RNA binding protein immunoprecipitation assays

Whole cell lysates were incubated with Rip buffer containing human anti-ago2 antibody or NC normal mouse IgG binding magnetic beads. Isolation of immunoprecipitated RNA was obtained by incubating the sample together with proteinase K. The concentration of RNA was measured by a spectrophotometer (NanoDrop; Thermo Scientific, Waltham, MA, USA). The quality of the RNA was evaluated by using a bioanalyzer (Agilent, Santa Clara, CA, USA). Furthermore, purified RNAs were extracted and analyzed by real-time qPCR to demonstrate the presence of the binding targets.

Ni^{2+}-NTA agarose bead pull-down assay

The SUMOylation of IGF2BP2 was analysed in HEK293T cells by using an in vivo SUMOylation assay with Ni^{2+}-NTA agarose beads. HA-IGF2BP2, His-SUMO1, His-SUMO2, His-SUMO3, Flag-Ubc9 and myc-SENP1 expressing plasmids were used to transfect HEK293T cells with LipofectamineTM 3000 (Invitrogen) according to the manufacturer's instructions. After 24h of cell transfection, HEK293T cells were collected. 10% of these cells were detected by Western blot; the rest cells were lysed in 1 ml of His-lysis buffer and then added to 20μl of Ni^{2+}-NTA agarose beads (Qiagen), and maintained on a rotation at 4°C overnight. The beads were successively washed (15 min per step) at room temperature with 1 ml in each of the following buffers: washing buffer-1 to -4. Finally, the beads were incubated in 60μl of elution buffer for 30 min at room temperature. The eluates were analyzed by western blotting. Buffers for Ni^{2+}-NTA agarose bead pull-down assay:

His-lysis buffer (guanidinium-HCl 6M, Na_2HPO_4/NaH_2PO_4 0.1M, Tris/HCl 0.01M, pH 8.0, imidazole 5mM, and β-mercaptoethanol 10mM); Washing buffer-1 (6M guanidinium-HCl, 0.1M Na2HPO4/NaH2PO4, 0.01M Tris/HCl, pH 8.0, 10mM β-mercaptoethanol); Washing buffer-2 (8M urea, 0.1M Na2HPO4/NaH2PO4, 0.01M Tris/HCl, pH 8.0, 10mM β-mercaptoethanol); Washing buffer-3 (6M guanidinium-HCl, 0.1M Na_2HPO_4/NaH_2PO_4, 0.01M Tris/HCl, pH 6.3, 5mM imidazole, and 10mM β-mercaptoethanol; 0.2% Triton X-100); Washing buffer 4 (6M guanidinium-HCl, 0.1M Na_2HPO_4/NaH_2PO_4, 0.01M Tris/HCl, pH 6.3, 5mM imidazole, and 10mM β-mercaptoethanol, 0.1% Triton X-100); Elution buffer (200mM imidazole, 0.15M Tris/HCl pH 6.7, 30% glycerol, 0.72M β-mercaptoethanol, 5% SDS).

Immunoprecipitation (IP)

Co-Immunoprecipitations were used to detected endogenous SUMOylation of IGF2BP2. Briefly, the RIPA (Beyotime Institute of Biotechnology) buffer were used to lyse the harvested cells (HEK293T, U87 and U251) on ice about 60 min and corresponding antibodies were incubated with the Protein A/G Magnetic beads for immunoprecipitation (Bimake) on ice for 30 min. Cell lysates were centrifuged, and the supernatants were added to and incubated with the protein A/G Magnetic beads (above incubated with corresponding antibodies) under rotation at 4 °C overnight. After be washed three times with high-salt buffer. The beads were boiled for 7 min with 2 × SDS sample buffer, followed by Western blot with corresponding antibodies. Immunoprecipitations were used to detected SUMOyaltion of IGF2BP2 effect ubiquitination. Cells co-transfected by HA-IGF2BP2-Wt or HA-IGF2BP2-3KR or His-SUMO1 or myc-SENP1 with or without Flag-Ub plasmids were lysed by RIPA buffer, and then subjected to immunoprecipitation, followed by immunoblotting with indicated antibodies.

Cell Counting Kit-8 (CCK-8) assay

The U87 and U251 glioma cells were plated into 96-well plates, 2000 cells per well. After 72 h of glioma cells (U87 and U251) transfection, 10μl of CCK-8 (CCK-8, Dojin, Japan) solution were added into each well and then incubated at 37 °C for 2 h. Use the SpectraMax M5 microplate reader (Molecular Devices, USA) to gauge the absorbance at 450 nm.

Cell transwell method

After preparing the cell suspension, suspended the cells in serum-free medium, count cells, and adjust the cell concentration at 1×105/ml level, then added 200μl of the prepared cell suspension to the upper chamber, while added 600μl culture medium of 10% serum to the lower chamber of the 24-well plate for the determination of the migration assay (or pre-coated with 500 ng/ml Matrigel solution (BD, Franklin Lakes, NJ, USA) for cell invasion assay). After incubation for 48 h, the chamber was removed and the cells on the membrane of the upper compartment were removed by physical methods. Then the chambers were fixed with methanol complementary with glacial acetic acid for 30 minutes and stained with 10% Giemsa (Dinguo, China). Three randomly areas were selected to count cells for statistics under a microscope and photographs were taken.

Reporter vectors construction and luciferase reporter assays

The sequence of OIP5-AS1 was amplified by PCR and cloned into pmirGLO Dual-luciferase miRNA Target Expression Vectors along with its mutant sequence of miR-495-3p binding sites

(GenePharama, Shanghai, China). Inoculation of HEK-293T cells in 96-well plates, when their inoculum reached 50-70%, and then the pmirGLO-OIP5-AS1 (or OIP5-AS1 mutant) reporter plasmid and agomiR-495-3p or antagomiR-495-3p NC plasmids were co-transfected into cells seeded into the 96-well plates. 48 hours after transfection, detection of luciferase activity by using the Prozyme Reporting System Kit (Promega). Renilla luciferase activity was obtained under standard calculations. The 3′-UTR sequence of HIF1A and MMP14 and their mutant sequence of miR-495-3p binding sites were cloned into pmirGLO Dual-luciferase miRNA Target Expression Vectors (GenePharama, Shanghai, China). Transfection procedures and calculation methods were similar to above.

Tumor xenograft implantation in nude mice

The nude mice were divided into three groups: Control group, IGF2BP2-Wt and IGF2BP2-3KR. The animals were free to autoclaved food and water during the experiment. 3×10^5 cells were subcutaneously injected in the right flanks of the mice. Tumor volume was measured every 5 days and the volume was calculated by the formula: volume (mm^3) = length width^2/2.44 days after injection, mice were sacrificed and tumors were isolated. For survival analysis in orthotopic inoculations, 3×10^5 cells were stereotactically implanted into the right striatum of the mice. The number of survived nude mice was recorded, and survival analysis was performed using Kaplan Meier survival curve.

Immunofluorescence staining

The cells grown on the surface of coverslips were fixed with 4% paraformaldehyde under the room temperature, followed by permeabilization with 0.2% Triton X-100, and then blocked with 10% goat serum in PBS. Next, coverslips were incubated with primary antibody diluted in 5% goat serum in PBS at 4°C overnight. The cells were washed three times with PBS and then incubated with fluorescent dye-conjugated secondary antibody diluted in 5% goat serum in PBS for 2h away from light. Furthermore, cells were washed three times with PBS and then stained with DAPI for 1h. The immunofluorescence images were recorded by a laser scanning confocal microscopy.

Cells VM formation assays

Each hole in the 96-well culture plate was covered with 100μl Matrigel Basement Membrane Matrix (BD Biosciences, Bedford, MA, USA). Meanwhile, the bubbles were avoided. The 96-well culture plate was incubated for 30 min in the incubator at 37°C. The cells were resuspended in 100μl of serum-free medium and seeded onto the surface of Matrigel at a density 6×10^5 cells/ml and incubated for 8 hours. The cells vascular structures were observed and photographed under an inverted microscope (Olympus, Tokyo, Japan). Three randomly areas were selected by an independent observer, and photographs were taken, the total number of tube-like structures per image were counted under a microscope for statistics.

CD34 endothelial marker periodic Acid-Schiff dual staining (CD34-PAS)

CD34-PAS was examined for the existence of VM. 5μm formalin-fixed and paraffin-embedded tissue specimens underwent dewaxed in xylene, hydrated in gradient ethanol and boiled in EDTA antigen-unmasking solution. After cooled gradually at room temperature, the tissue specimens were incubated with peroxide, blocked with goat serum and incubated with CD34 primary monoclonal

antibody (1:50, Proteintech, Rosemont, IL) at 4°C for 16h. After incubation with secondary antibody at 37°C for 10 min, the tissue specimens were stained with DAB kit (MaiXin Biotech, China). Periodic acid solution, schiff solution and hematoxylin were used for the next PAS staining. VM density was counted under microscope in five random fields.

Supplementary Figure 1

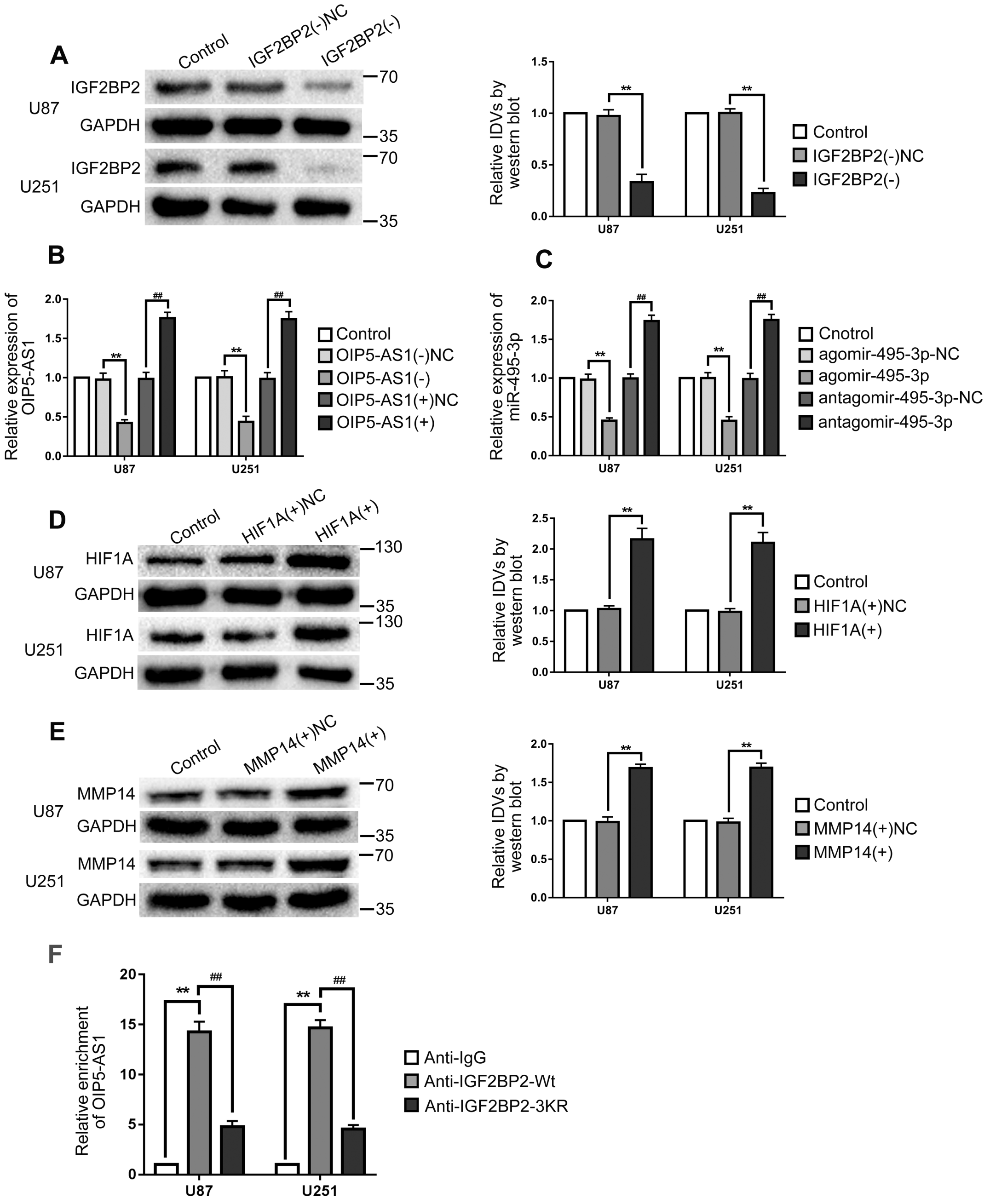

Figure S1 Transfection efficiency of IGF2BP2, OIP5-AS1, miR-495-3p, HIF1A and MMP14 in glioma cells (U87 and U251)

(A) Relative protein expression of IGF2BP2 knockdown in U87 and U251 cells. Data are presented as mean ± SD (n=3). **P< 0.01, vs. IGF2BP2(-)NC. **(B)** Relative mRNA expression of knockdown and overexpression of OIP5-AS1 in U87 and U251 cells. Data are presented as mean ± SD (n=3). **P< 0.01, vs. OIP5-AS1(-)NC, ##P< 0.01, vs. OIP5-AS1(+)NC. **(C)** Relative mRNA expression of miR-495-3p agomir or antagomir in U87 and U251 cells. Data are presented as mean ± SD (n=3). **P< 0.01, vs. agomiR-495-3p NC, ##P< 0.01, vs. antagomiR-495-3p NC. **(D)** Relative protein

expression of HIF1A overexpression in U87 and U251 cells. Data are presented as mean ± SD (n=3). **P< 0.01, vs. HIF1A(+)NC. **(E)** Relative protein expression of MMP14 overexpression in U87 and U251 cells. Data are presented as mean ± SD (n=3). **P< 0.01, vs. MMP14(+)NC. **(F)** Verifying the binding interaction between IGF2BP2-Wt/IGF2BP2-3KR and OIP5-AS1. Data are expressed as the mean ± SD (n=3). **p<0.01 vs. anti-IgG. ##p<0.01 vs. anti-IGF2BP2-Wt.
